# Glucose-6-phosphate dehydrogenase is indispensable in embryonic development by modulation of epithelial-mesenchymal transition via the NOX/Smad3/miR-200b axis

**DOI:** 10.1038/s41419-017-0005-8

**Published:** 2018-01-09

**Authors:** Yi-Hsuan Wu, Ying-Hsuan Lee, Hung-Yu Shih, Shih-Hsiang Chen, Yi-Chuan Cheng, Daniel Tsun-Yee Chiu

**Affiliations:** 1 Research Center for Chinese Herbal Medicine, College of Human Ecology, Chang Gung University of Science and Technology, Taoyuan, Taiwan; 2 Department of Medical Biotechnology and Laboratory Science, College of Medicine, Chang Gung University, Taoyuan, Taiwan; 3 Graduate Institute of Biomedical Sciences, College of Medicine, Chang Gung University, Taoyuan, Taiwan; 4 Department of Pediatric Hematology/Oncology, Linkou Chang Gung Memorial Hospital, Taoyuan, Taiwan; 5 Neuroscience Research Center, Chang Gung Memorial Hospital at Linkou Medical Center, Taoyuan, Taiwan; 6 Healthy Aging Research Center, Chang Gung University, Taoyuan, Taiwan

## Abstract

Glucose-6-phosphate dehydrogenase (G6PD) is a housekeeping enzyme involved in the pentose phosphate shunt for producing nicotinamide adenine dinucleotide phosphate (NADPH). Severe G6PD deficiency leads to embryonic lethality, but the underlying mechanism is unclear. In the current study, the effects of G6PD on epithelial–mesenchymal transition (EMT), especially during embryonic development, were investigated. The knockdown of *G6PD* induced morphological changes, accompanied by the suppression of epithelial markers, E-cadherin and β-catenin, in A549 and MDCK cells. Such modulation of EMT was corroborated by the enhancement of migration ability in *G6PD*-knockdown A549 cells. Zebrafish embryos with *g6pd* knockdown exhibited downregulation of the E-cadherin/β-catenin adhesion molecules and impaired embryonic development through reduction in epiboly rate and increase in cell shedding at the embryo surface. The dysregulation in zebrafish embryonic development caused by *g6pd* knockdown could be rescued through human *G6PD* or *CDH1* (E-cadherin gene) cRNA coinjection. The Smad3/miR-200b axis was dysregulated upon *G6PD* knockdown, and the reconstitution of *SMAD3* in *G6PD*-knockdown A549 cells restored the expression of E-cadherin/β-catenin. The inhibition of NADPH oxidase (NOX) activation through the loss of p22_phox_ signaling was involved in the dysregulation of the Smad3/miR-200b axis upon *G6PD* knockdown. The reconstitution of *G6PD* led to the recovery of the regulation of NOX/Smad3/miR-200b signaling and increased the expression of E-cadherin/β-catenin in *G6PD*-knockdown cells. Thus, these results suggest that in the EMT process, G6PD plays an important regulatory role as an integral component of the NOX/Smad3/miR-200b axis.

## Introduction

Glucose-6-phosphate dehydrogenase (G6PD) is a housekeeping enzyme with the major function of regenerating nicotinamide adenine dinucleotide phosphate (NADPH) to maintain cellular redox homeostasis^1–4^. Because NADPH is essential for NADPH oxidase (NOX) and nitric oxide synthase to produce reactive oxygen and nitrogen species for signaling, many new cellular roles of G6PD have been identified. G6PD modulates xenobiotic metabolism via the Nrf2 signaling pathway and affects the xenobiotic metabolizing enzyme expression^5^. Moreover, it acts as a key regulator of the cellular inflammatory and immune response against viral infection^6,7^. Because G6PD is a rate-limiting enzyme in the pentose-phosphate shunt, it is essential for regulating energy consumption and glucose metabolism^8^. These studies have indicated that G6PD plays an important role in modulating many cellular functions.

Severe G6PD deficiency is embryonically lethal^9,10^. G6PD is essential in the regulation of embryonic development. Severe G6PD deficiency in mice causes the demise of male embryos in hemizygous G6PD deficiency and female embryos in heterozygous G6PD deficiency^9^. Reduced litter size is also observed in mice with slight G6PD deficiency^11^. The study of a severe G6PD deficiency mouse model delineates that the initial development of all embryos, especially in early gestation, is unimpaired^9^. In *Caenorhabditis elegans*, G6PD deficiency has a strong impact on egg hatching and embryonic development; this is attributed to altered lipid metabolism^12^. All these data indicate that G6PD is vital in embryonic development and differentiation. However, the underlying mechanism through which G6PD affects embryonic development has not fully been elucidated.

A possible mechanism for G6PD to modulate embryonic development could be mediated by its effect on epithelial–mesenchymal transition (EMT). EMT is a process in which cells transit between the epithelial and mesenchymal states and drives many important aspects of embryonic development^13^. It is characterized by the loss of epithelial surface markers, which occurs in response to EMT transcription factors or epigenetic regulators. Transforming growth factor β (TGF-β) is vital because of its abilities in the molecular regulation of EMT for the development of metazoans^14,15^. It is temporally expressed in the developing heart of mice^16^, and its subfamilies are the key regulators for the generation of axes during embryogenesis^17^. Data from the Gene Expression Omnibus database indicate that the gene expression of *G6PD* is downregulated in TGF-β-treated cells (accession nos. GDS3710, GDS2975, and GDS4106). This implies that G6PD may be a factor in regulating the EMT process. The relationship between the G6PD status and EMT process thus warrants investigation.

In the current study, G6PD was identified for the first time as a regulator of the EMT process during embryonic development, demonstrated by cell lines and zebrafish models. *G6PD* knockdown modulated the expression of EMT markers and zebrafish embryonic development. The modulation of EMT through *G6PD* knockdown is correlated with the impairment of NOX/Smad3/miR-200b signaling. These data suggest that G6PD plays an important role in regulating embryonic development, as an integral part of the NOX/Smad3/miR-200b axis for modulating the expression of adhesion molecules.

## Results

### EMT-associated morphological changes in *G6PD*-knockdown A549 and MDCK cells

**Fig. 1:**
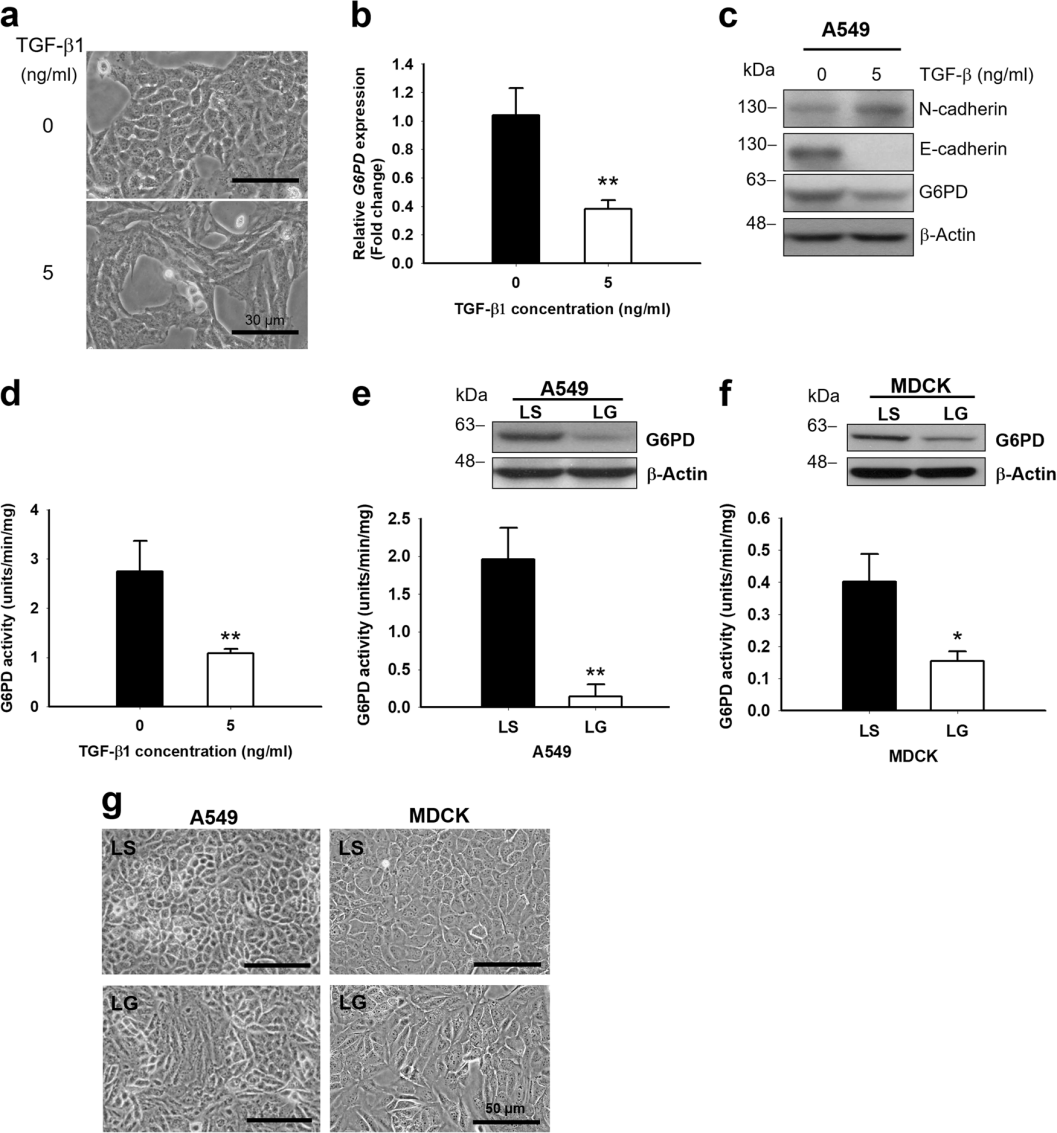
*G6PD* knockdown induced morphological changes in epithelial cells **a** TGF-β treatment induced morphological changes in A549 cells, accompanied by G6PD inhibition. A549 cells were serum starved for 24 h and treated with 5 ng/mL of TGF-β for 24 h. Compared with the control cells, A549 cells exhibited constricted and mesenchymal-like morphologies after TGF-β stimulation (5 ng/mL). Scale bar, 30 µm. One representative example is shown out of three experiments. **b** The transcript level of *G6PD* in TGF-β-treated A549 cells was analyzed through real-time PCR. The relative result was normalized to *ACTB*, calculated relative to that without the TGF-β treatment counterpart (set to 1 for one experiment), and presented as mean ± SD (**p < 0.01) by performing independently in triplicate. **c** Protein expressions of G6PD, E-cadherin, and N-cadherin in A549 cells with or without TGF-β stimulation were analyzed through Western blotting. β-Actin was used as the loading control. One representative example is shown out of three experiments. **d** G6PD activities were determined in A549 cells with or without TGF-β stimulation. Results are expressed as mean ± SD (**p < 0.01). **e** G6PD expressions and activities of G6PD-knockdown (LG) A549 and control (LS) cells were determined through Western blot and G6PD activity assay. Results are expressed as mean ± SD (**p < 0.01). One representative example is shown out of three western blot experiments. **f** G6PD expressions and activities of G6PD-knockdown (LG) MDCK and control (LS) cells were determined through Western blot and G6PD activity assay. Results are expressed as mean ± SD (*p < 0.05). One representative example is shown out of three Western blot experiments. **g** A549-LG and MDCK-LG cells exhibited constricted and mesenchymal-like morphologies compared with LS cells. Scale bar, 50 µm. One representative example is shown out of four experiments

To ascertain whether EMT progression is linked to *G6PD* expression, A549 cells treated with TGF-β were used as cell model. After TGF-β1 treatment for 24 h, a clear morphological change from epithelial (round shape) to mesenchymal (spindle shape) cell types (Fig. 1a) was observed in A549 cells, with E- to N-cadherin switching by decreasing E-cadherin and increasing N-cadherin expression (Fig. 1c). Meanwhile, a decrease in the transcriptional and translational levels of G6PD was detected through polymerase chain reaction (PCR), Western blot, and G6PD activity assays (Fig. 1b, c, and d). To assess whether the morphological differences were associated with cellular G6PD expression, we knocked down *G6PD* by using lentiviruses to deliver shRNA into two epithelial cell lines, namely A549 and MDCK cells. *G6PD*-knockdown efficiency was confirmed through protein expression and activity assay (Figs. 1e-f). Compared with the control A549-LS (Lentiviral Scramble control) cells, A549-LG (Lentiviral G6PD shRNA) cells expressed approximately 16% G6PD levels (Fig. 1e). MDCK-LG cells expressed 42% G6PD levels compared with the control MDCK-LS cells (Fig. 1f). *G6PD*-knockdown cells presented significant alteration in cellular morphology, and both A549-LG and MDCK-LG cells exhibited more mesenchymal-like morphology than did their control cells (Fig. 1g).

### Decreased expressions of E-cadherin and β-catenin through *G6PD* knockdown in A549 and MDCK cells

**Fig. 2:**
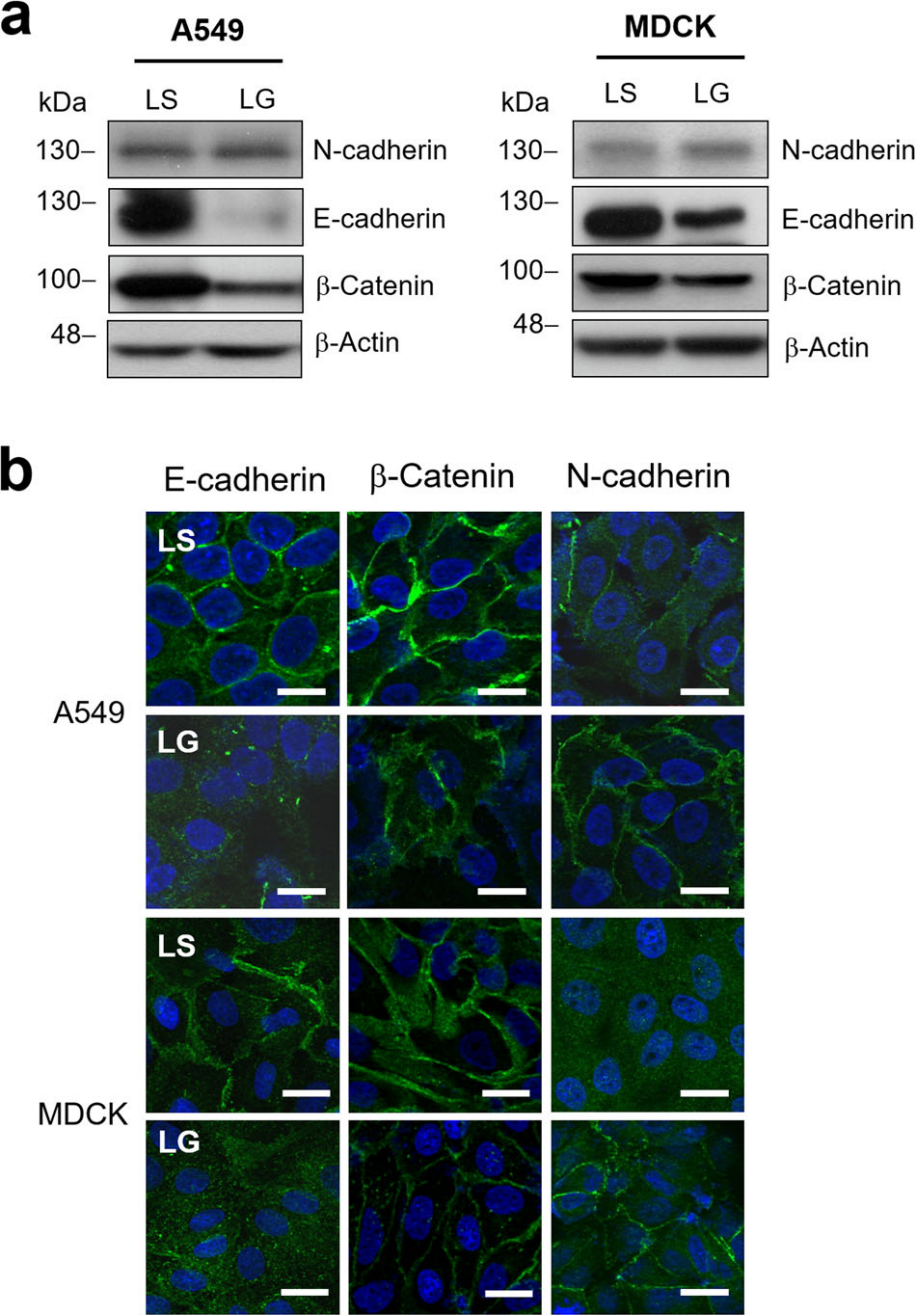
The expression of EMT adhesion molecules were altered by the G6PD status in A549 and MDCK cells **a** Compared with control (LS) cells, a significant decrease in the protein expressions of E-cadherin and β-catenin and an increase in N-cadherin expression in A549-LG and MDCK-LG cells were observed through Western blot analysis. β-Actin was used as the loading control. One representative example is shown out of three experiments. **b** Immunofluorescence staining for E-cadherin, N-cadherin, and β-catenin (left, right, and central panels, respectively; green) was observed in A549-LG and MDCK-LG cells compared with in their control LS cells. Nuclei were stained with Hoechest 33342 (blue*)*. Scale bar, 20 µm. One representative example is shown out of three experiments

To further investigate the correlation between G6PD and EMT, the EMT-associated proteins were analyzed in *G6PD*-knockdown A549 and MDCK cells. The downregulation of E-cadherin and 8–10% upregulation of N-cadherin were observed in A549-LG and MDCK-LG cells (Fig. 2a). The expression of β-catenin, a subunit of the cadherin protein complex, was also decreased in A549-LG and MDCK-LG cells (Fig. 2a). Moreover, compared with the respective control cells, a reduction in E-cadherin and β-catenin expression and increase in N-cadherin expression at the cell surface were also observed in A549-LG and MDCK-LG cells examined through immunofluorescent staining (Fig. 2b). Using siRNA to further confirm our observation, the gene expression of *CDH1* and *CTNNB1* were downregulated upon *G6PD* knockdown concomitant with *CDH2* and *SNAI1* upregulation in A549 cells (Supplementary Figure 1). These results demonstrate that the inhibition of G6PD expression can reduce cell–cell adhesion through the dysregulation of the adhesion protein complex, which leads to the modulation of EMT.

### Enhanced cell migration in *G6PD*-knockdown A549 cells

**Fig. 3:**
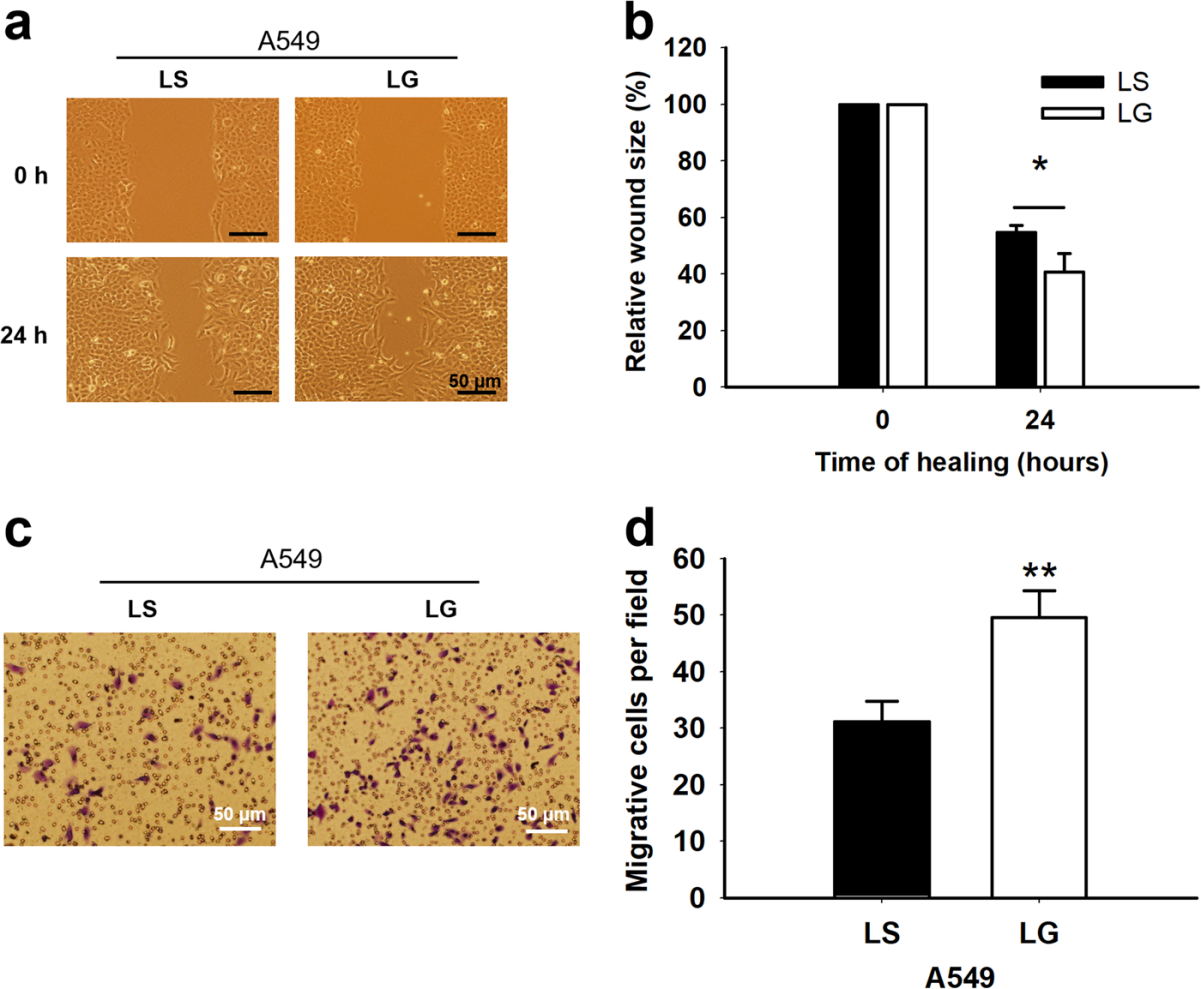
Enhanced motility was observed in A549-LG cells **a** Wound-healing assay was performed to analyze the motility of A549-LS and -LG cells. After serum starvation for 24 h, cell mono-layers were wounded with a sterile 200-µL tip and washed with a serum-free medium. Cells were photographed at 0 h and incubated in Dulbecco’s modified Eagle’s medium (DMEM) with 1% fetal bovine serum (FBS) for 24 h. Images represent the results obtained from four independent experiments. **b** Quantification was performed by measuring the size of the wound at indicated time points. The size at 24 h was calculated relative to that at 0 h (set to 100%), and the graph shows the relative wound size in A549-LS cells and A549-LG cells (*p < 0.05). **c** In vitro migration assay was performed to analyze the motility of A549-LS and -LG cells. Cell mono-layers were seeded on Millicell® culture plate inserts (pore size, 8 μm; Millipore) in serum-free DMEM, and they migrated into the lower compartment containing DMEM with 10% FBS. The migrated cells were fixed, stained, and photographed at 24 h. Scale bar, 50 µm. Images represent the results obtained from five independent experiments. **d** Quantification was conducted by counting migrated cells in five different fields of each independent experiment, and the results are presented as mean ± SD (**p < 0.01)

Because the downregulation of E-cadherin and β-catenin is often associated with increased cell migration, a scratch wound assay was performed for 24 h. *G6PD* knockdown was accompanied by enhanced cell migration by 31 ± 3.6% in A549-LG cells compared with A549-LS cells (Figs. 3a, b, *p* < 0.05; raw data in Supplementary Table 1). Furthermore, to exclude the effect of proliferation caused by *G6PD* knockdown, we performed a transwell migration assay. Inhibiting G6PD expression increased migration over a membrane by 59 ± 3.7%, compared with the A549-LS group (Figs. 3c, d, *p* < 0.05). These enhanced migration data provide further support to the notion that *G6PD* knockdown can affect EMT.

### Impaired embryonic development of zebrafish by *g6pd* knockdown

**Fig. 4:**
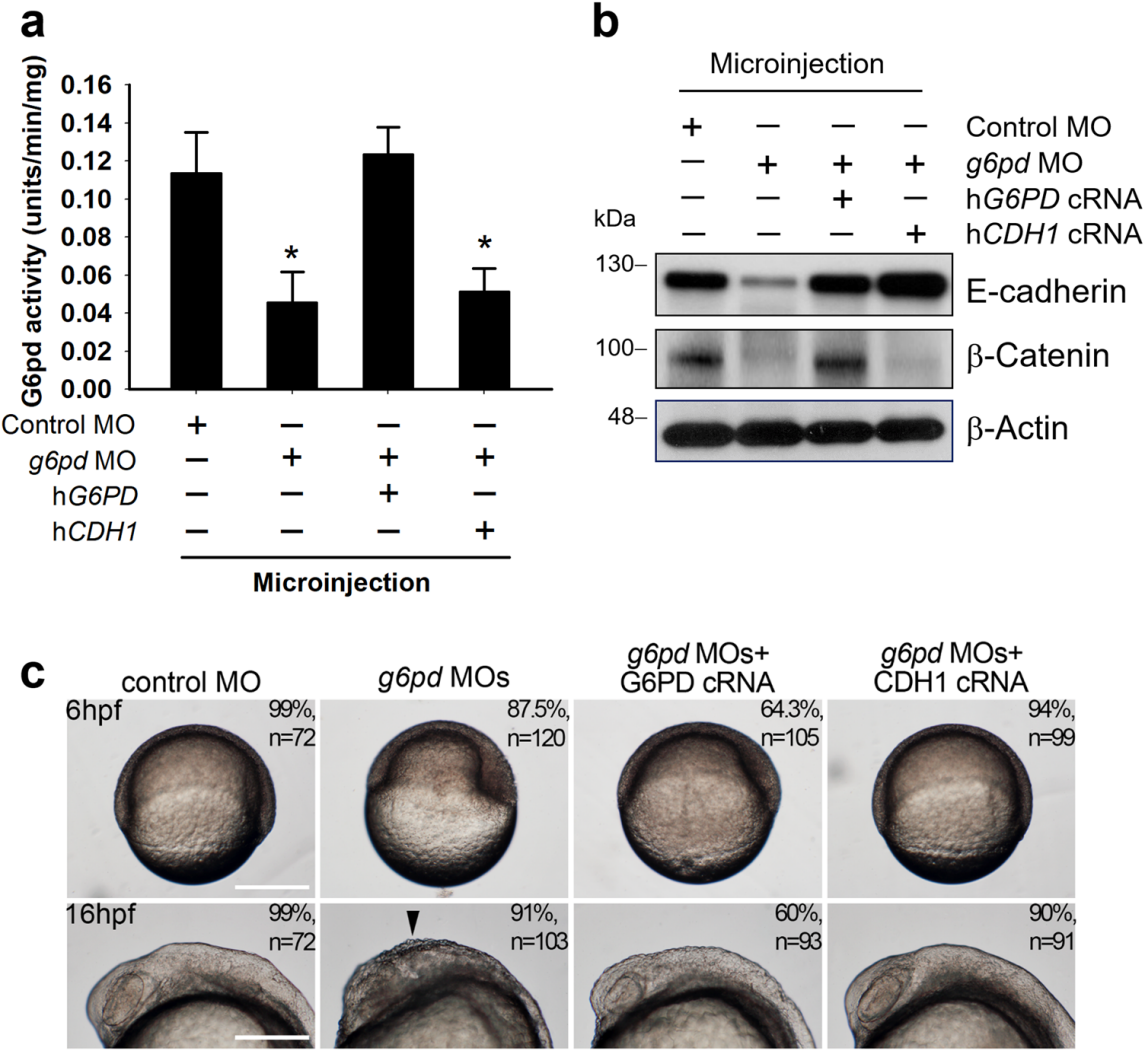
Knockdown of *g6pd* in embryos decreased the expression of E-cadherin in zebrafish **a** Single-cell zebrafish embryos were injected with random MO, MOs targeting *g6pd* only, and MOs targeting *g6pd* with human *G6PD* or human *CDH1* cRNA rescue. Whole-cell lysates were prepared at 72 hpf from embryo injection. G6pd activities were determined through the G6pd activity assay, as described in the Materials and Methods section. Results are expressed as mean ± SD (*p < 0.05 compared with the control MO group). **b** Protein expressions of E-cadherin and β-catenin in the whole-embryo lysates at 72 hpf were analyzed through Western blot. β-Actin was used as the loading control. One representative Western blot example is shown out of three experiments. **c** Zebrafish embryos were injected with random MO, MOs targeting *g6pd* only, or MOs targeting *g6pd* with human *G6PD* or human *CDH1* cRNA rescue and were monitored through microscopy. Gastrulation was initiated in the MO-injected embryos at 6 hpf. The epiboly rate was decreased in the *g6pd* MO-injected embryos (top panel). Scale bar, 250 μm. The phenotypic examination of *g6pd* MO at 16 hpf revealed shedding of the cells at the embryo surface (arrowhead in in the bottom panel). Rescue of the phenotype was performed by coinjecting *g6pd* MOs with human *G6PD* or human *CDH1* cRNA. n, total number of embryos analyzed from three independent experiments. Scale bar, 250 μm

Because EMT plays an important role during embryonic development^14,15^, the effects of *g6pd* knockdown on early embryonic development were investigated in a zebrafish model. The optimal delivering dose of *g6pd* morpholino (MO) were determined following Lund’s group^18^. The kinetics of G6pd activity at 24, 48, and 72 h postfertilization (hpf) are shown in Supplementary Figure 2. The kinetic data of control MO revealed that G6pd activity was increased during zebrafish embryonic development (Supplementary Figure 2a). However, *g6pd* MO injection reduced G6pd activity (Supplementary Figure 2a). Compared with the control embryos at 72 hpf, the embryos injected with *g6pd* MO exhibited a decrease in G6pd activity (41% of control MO; *p* < 0.05; Fig. 4a) and the inhibition of E-cadherin and β-catenin expression (Fig. 4b). Moreover, the injection of human *G6PD* cRNA restored the G6pd activity and the expression of E-cadherin and β-catenin in the *g6pd* morphants (Figs. 4a, b). The expressions of EMT markers were also altered by the G6pd status in zebrafish embryos even at 6 and 16 hpf (Supplementary Figure 3). Decreased expression of β-catenin in *g6pd* MO embryos could be rescued through the coinjection of human *G6PD* cRNA but not *CDH1* cRNA (Fig. 4b).

E-cadherin is involved in the regulation of zebrafish cell movement and tissue formation during early embryogenesis where loss of E-cadherin decreases epiboly rate and reduces cell–cell adhesion^19^. Additional phenotype experiments were performed to determine whether other EMT-related developmental defects could be observed in the *g6pd*-knockdown embryos. Indeed, the epiboly rate was decreased (Fig. 4c, up panel), and the change of cell shape at the embryo surface was also observed (Fig. 4c, bottom panel) in *g6pd* MO-injected embryos compared to control embryos at 6 and 16 hpf, respectively. The coinjection of *g6pd* MO with human *G6PD* or *CDH1* cRNA rescued these developmental defects (Fig. 4c). All these data indicate that *g6pd* knockdown influenced the embryonic development of zebrafish through the dysregulation of E-cadherin and β-catenin expression, which led to defective EMT.

### Downregulation of E-cadherin and β-catenin through *G6PD* knockdown via the inhibition of the Smad3/miR-200b pathway

**Fig. 5:**
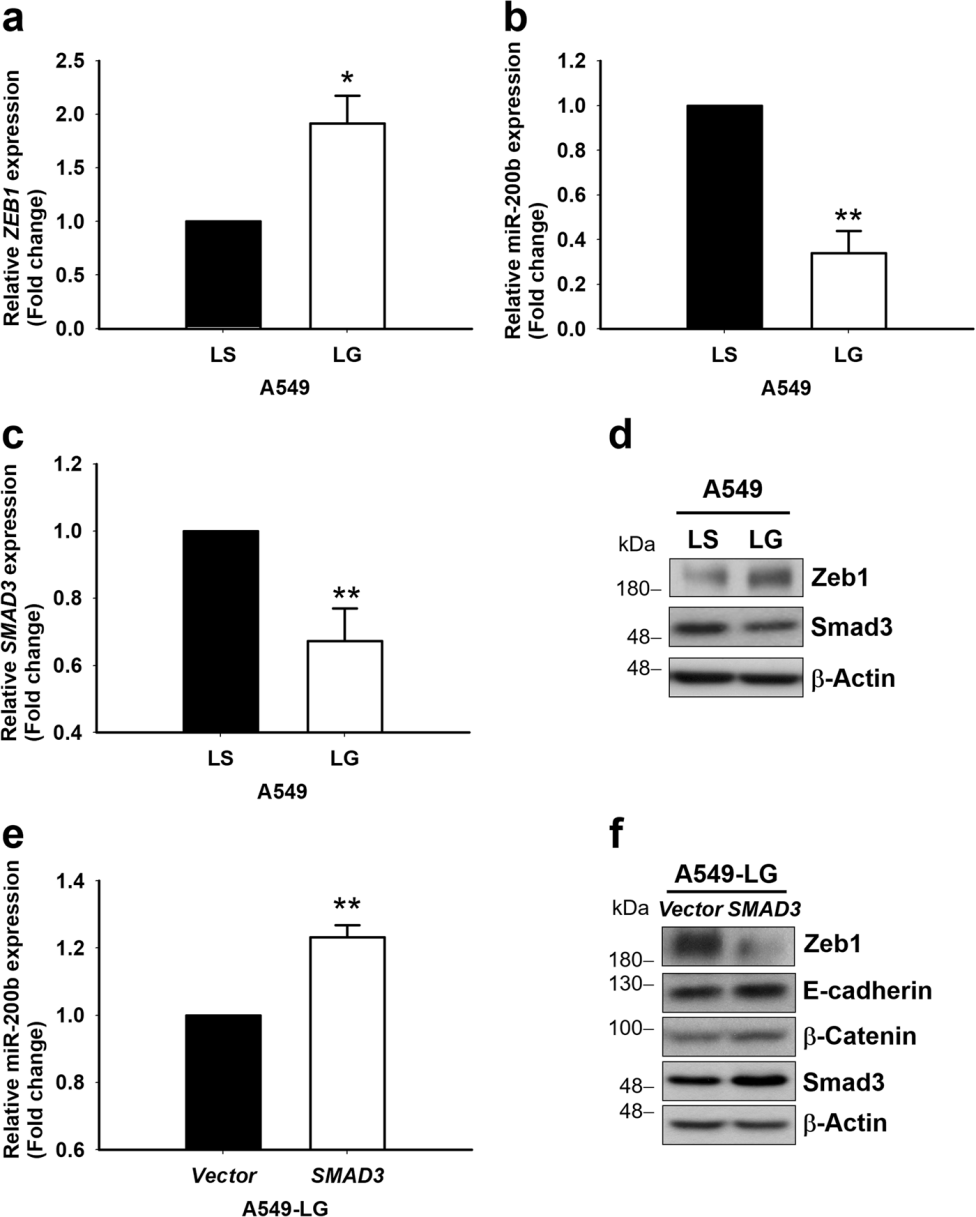
Inhibition of G6PD downregulated the expression of miR-200b via the Smad3 pathway in A549 cells **a** The transcript level of *ZEB1* was determined through real-time PCR, normalized to *ACTB*, and calculated relative to the LS group (set to 1). Results are presented as mean ± SD from three independent experiments (*p < 0.05). **b** The transcript levels of miR-200b in A549-LS and -LG cells were validated through real-time PCR and normalized to *RNU6*. The expression level was calculated relative to LS (set to 1) and presented as mean ± SD from three independent experiments (**p < 0.01). **c** The transcript level of *SMAD3* was determined through real-time PCR, normalized to *ACTB*, and calculated relative to the LS group (set to 1). Results are presented as mean ± SD from three independent experiments (**p < 0.01). **d** Protein lysates collected from A549-LG and -LS cells were used to analyze the expressions of Zeb1 and Smad3. β-Actin was used as the loading control. One representative example is shown out of three experiments. **e** Ectopic Smad3 was expressed in *SMAD3*-overexpressing A549-LG cells, and the transcript levels of miR-200b were analyzed through real-time PCR, normalized to *RNU6*, and calculated relative to the vector control (set to 1). Data are presented as mean ± SD from three independent experiments (**p < 0.01). **f** The protein expressions of Smad3, E-cadherin, β-catenin, and Zeb1 were analyzed in *SMAD3*-overexpressing A549-LG cells. β-Actin was used as the loading control. One representative example is shown out of three experiments

Upon delineating the possible mechanism through which G6PD affects E-cadherin expression, Zeb1, a well-known transcription inhibitor of E-cadherin, was examined. The result showed Zeb1 was upregulated in A549-LG cells compared with in A549-LS cells (Fig. 5a, d). Further study of the miR-200 family, the regulatory miRNAs targeting *ZEB1* and modulating the expression of β-catenin^20^, revealed that miR-200b was downregulated in A549-LG cells (Fig. 5b). Moreover, the transcriptional (Fig. 5c) and translational levels (Fig. 5d) of Smad3, the upstream regulator of miR-200b, were also downregulated in A549-LG cells. The reconstitution of *SMAD3* in A549-LG cells increased endogenous miR-200b expression (Fig. 5e), downregulated Zeb1 expression, and upregulated the expressions of E-cadherin and β-catenin (Fig. 5f). These results indicate that *G6PD* knockdown functionally modulates EMT by inhibiting the Smad3/miR-200b pathway.

### Upstream mechanism for EMT modulation through *G6PD* knockdown due to NOX inhibition

**Fig. 6:**
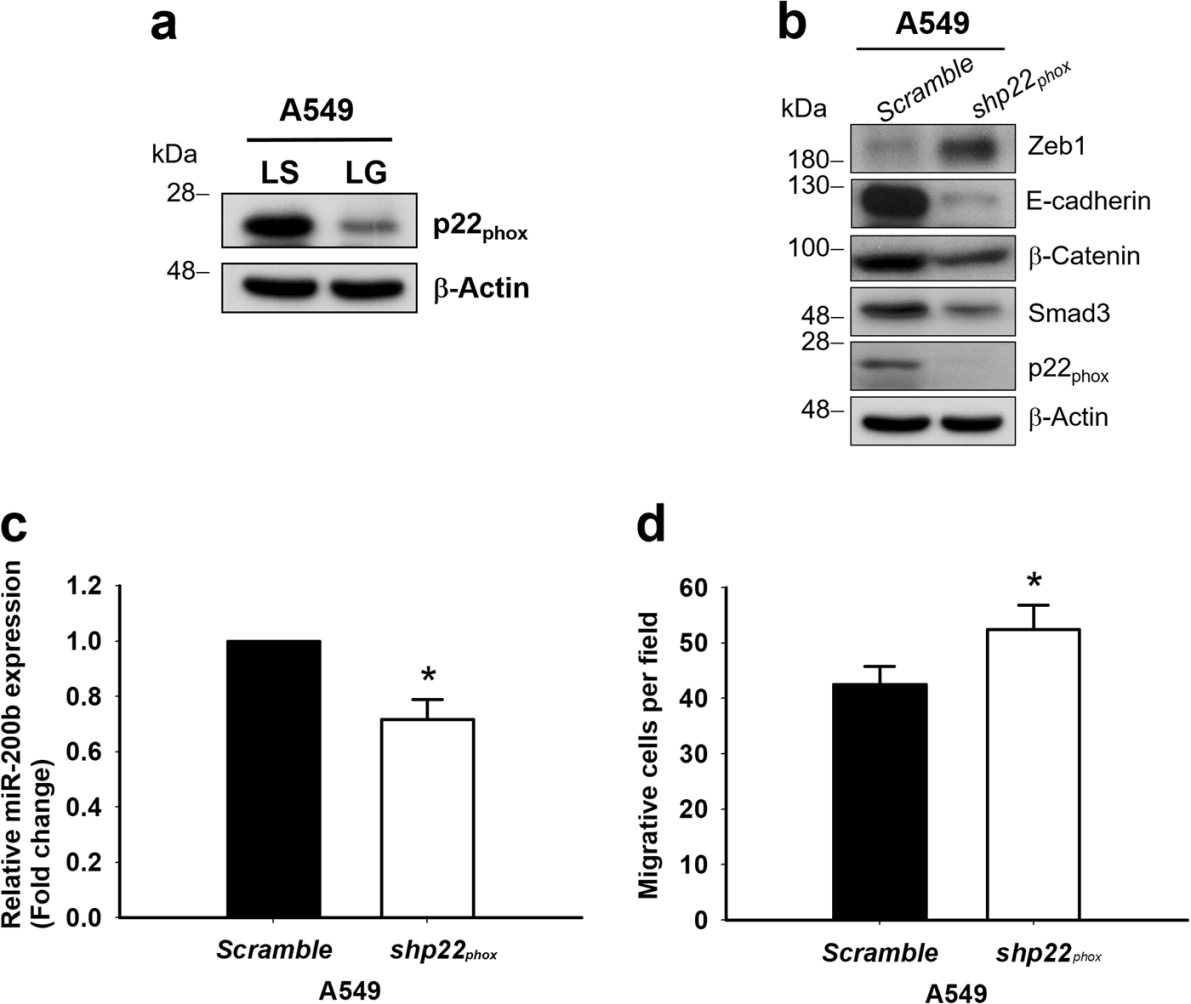
Decreased expression of Smad3 in A549-LG cells was due to impaired NOX function **a** The protein level of p22_phox_ was determined through Western blot analysis in A549-LS and LG cells. β-Actin was used as the loading control. One representative example is shown out of three experiments. **b** A549 cells were transducted with recombinant lentivirus for knocking down *p22*_*phox*_. Ninety-six hours later, the cell lysate was harvested for analysis. The protein expressions of p22_phox_, Smad3, Zeb1, E-cadherin, and β-catenin were measured through Western blot. β-Actin was used as the loading control. One representative example is shown out of three experiments. **c** The level of miR-200b expression was determined in *p22*_*phox*_-knockdown and control A549 cells through real-time PCR. Data are presented as mean ± SD (*n* = 3). *p < 0.05. **d** In vitro migration assay was performed to analyze the motility of *p22*_*phox*_-knockdown and control A549 cells. Quantification was conducted by counting migrated cells in five different fields of each independent experiment, and the results are presented as mean ± SD (*p < 0.05)

Because NOX activation is the key regulator of the Smad3 pathway, the mechanism of the G6PD status in the Smad3 pathway was delineated by analyzing the expressions of NOXs in these cells. The mRNA expression levels of *NOX1*, *NOX2*,* p22*_*phox*_,* and p67*_*phox*_, the major NOX subunits in A549 cells, were downregulated in A549-LG cells (data not shown). Because p22_phox_ is the common subunit of NOX1 and NOX2, we further analyzed the protein expression of p22_phox_ in A549-LS and LG cells. As shown in Fig. 6a, the translational level of p22_phox_ was downregulated in A549-LG cells compared with in A549-LS cells. Further study was performed by knocking down *p22*_*phox*_ in A549 cells, and decreased protein expressions of Smad3, E-cadherin, and β-catenin, along with upregulated Zeb1, were observed (Fig. 6b). The *SNAI1* gene expression was upregulated by *p22*_*phox*_ knockdown (Supplementary Figure 4). The level of miR-200b was also downregulated in *p22*_*phox*_-knockdown A549 cells (Fig. 6c), and increased migration over a membrane by 23 ± 5.7%, compared with the scramble control group (Fig. 6d, *p* < 0.05). These data indicate that the modulation of the EMT process through *G6PD* knockdown may partly occur due to the downregulation of the NOX/Smad3/miR-200b pathway.

### Increased E-cadherin/β-catenin expression by *G6PD* reconstitution in A549-LG cells through NOX/Smad3/miR-200b signaling

**Fig. 7:**
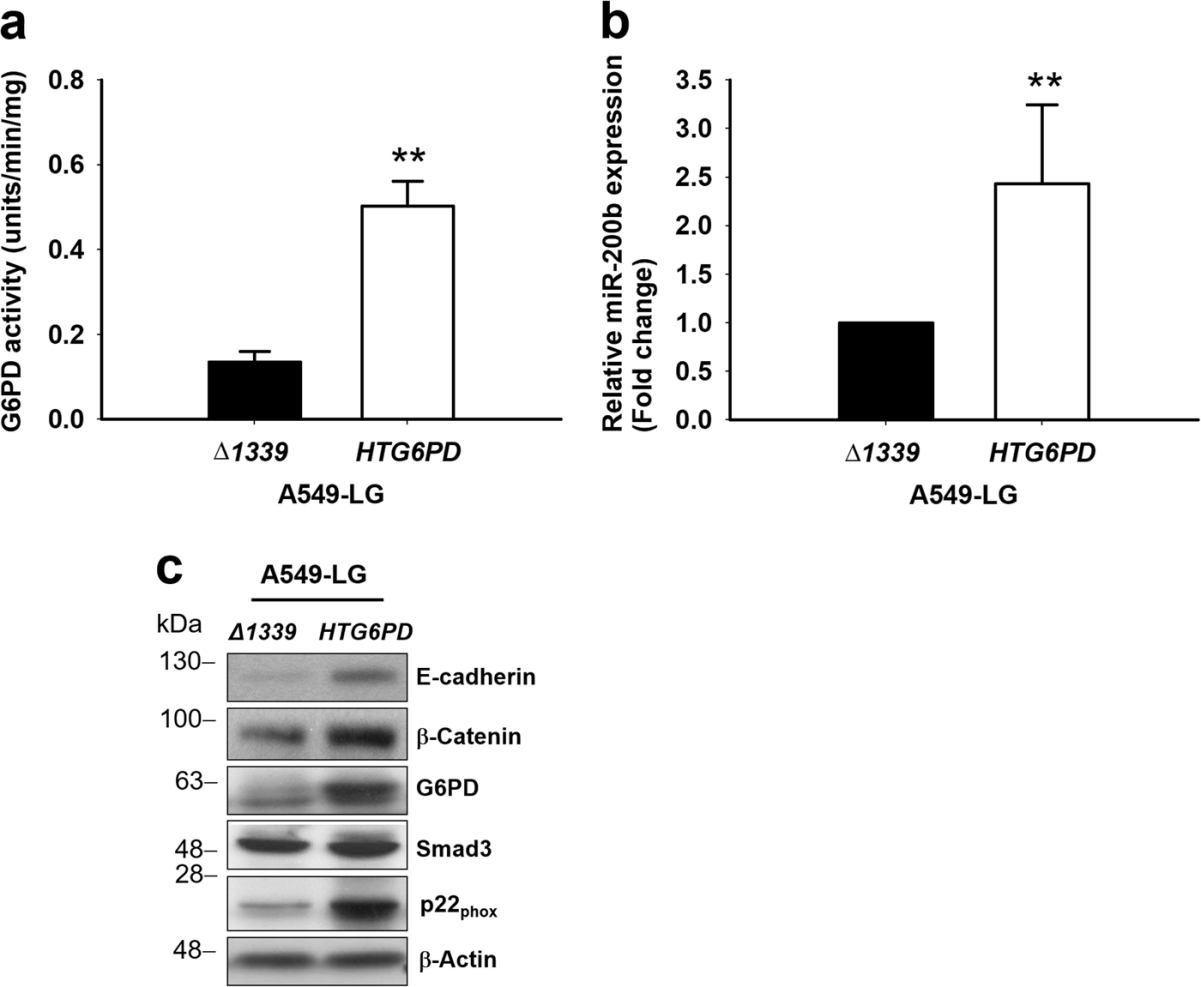
G6PD reconstitution in A549-LG cells repaired the protein expressions of the NOX/Smad3/miR-200b axis and increased E-cadherin/β-catenin expression **a** Protein extracts (10 µg) of G6PD-reconstituted A549-LG (HTG6PD) and negative control (Δ1339) cells were used to determine G6PD activities. Results are expressed as mean ± SD from three independent experiments (**p < 0.01). **b** The transcript level of miR-200b was measured through real-time PCR, normalized to *RNU6*, and calculated relative to the Δ1339 group (set to 1). Results are presented as mean ± SD from three independent experiments (**p < 0.01). **c** The protein expressions of G6PD, E-cadherin, β-catenin, P22_phox_, and Smad3 were determined in *HTG6PD* and Δ1339-transduced A549-LG cells. β-Actin was used as the loading control. One representative example is shown out of three experiments

We further reconstituted *G6PD* expression in A549-LG cells, using Δ1339 as the dominant negative control (1339G>A, classic I G6PD deficiency). The transfection of *HTG6PD* increased the G6PD activity in A549-LG cells, and the transfection of Δ1339 in A549-LG cells did not affect the G6PD activity (Fig. 7a). MiR-200b expression was highly correlated with G6PD activity (Fig. 7b), and G6PD reconstitution upregulated E-cadherin and β-catenin expression and increased the expression of Smad3 and p22_phox_ (Fig. 7c). Moreover, E-cadherin expression could be downregulated by miR-200b LNA and upregulated by ZEB1 siRNA even in the presence of Δ1339 or *HTG6PD* A549-LG cells (Supplementary Figure 5). Together, these data provide novel information concerning the role of G6PD in modulating the EMT process through the NOX/Smad3/miR-200b pathway.

## Discussion

G6PD is a well-known housekeeping enzyme whose major function is to maintain cellular redox homeostasis^12,21,22^, and studies in knockout mouse reveals that G6PD plays a fundamental role in embryonic development. However, the detailed mechanism regarding why its knockout is embryonically lethal has not been clearly elucidated. The evidence obtained in the current study indicates that G6PD plays an important role in embryonic development, partly by affecting the expression of adhesion molecules. *G6pd* knockdown in zebrafish can cause EMT or adhesion defects during embryonic development, whereas *CDH1* cRNA coinjection can rescue such phenotype. Moreover, *G6PD* knockdown modulates the EMT process by impairing NOX/Smad3/miR-200b signaling. These findings suggest a possible mechanism through which G6PD status affects embryonic development (Fig. 1).

**Fig. 8:**
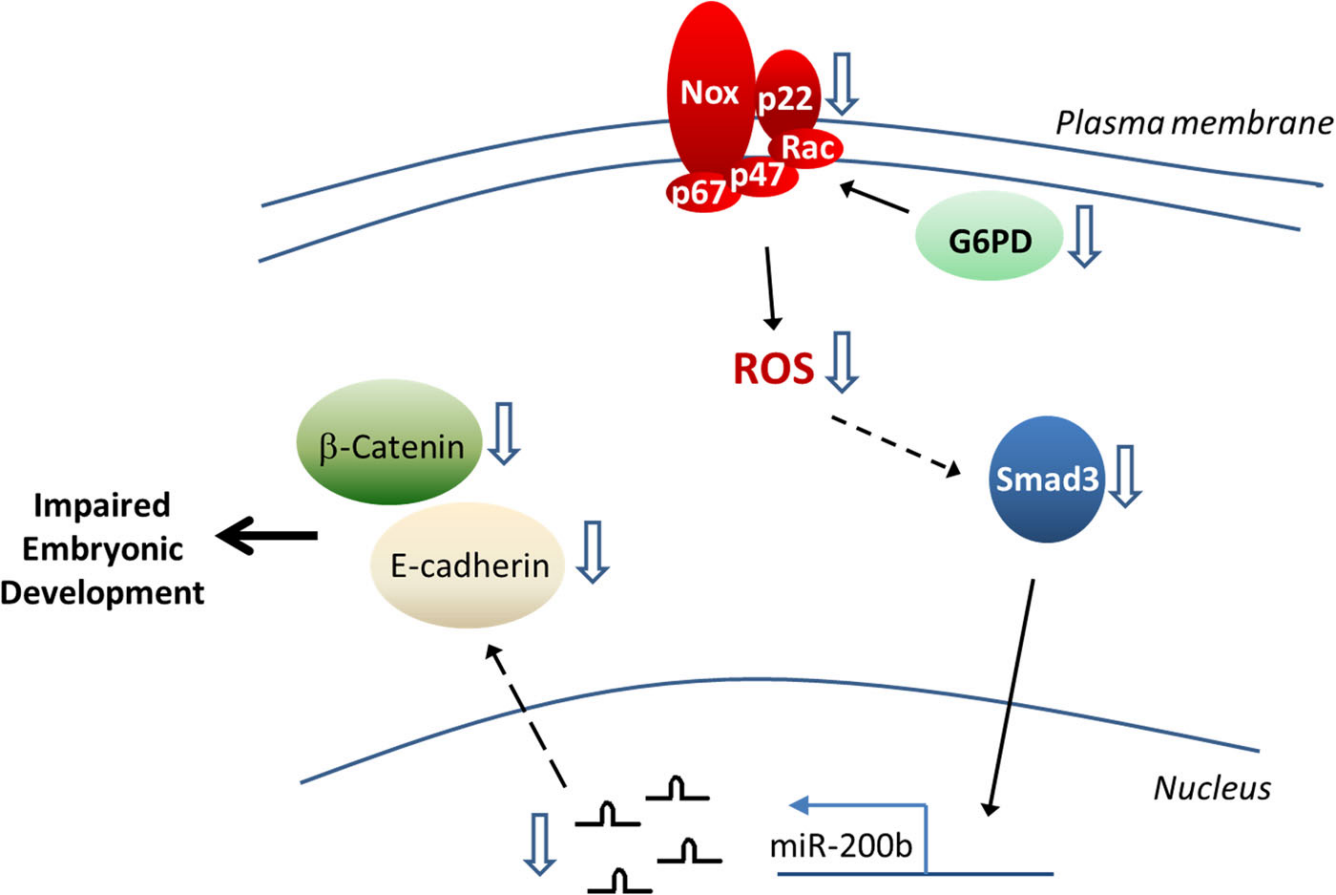
Schematic depicting how G6PD status modulates the expressions of adhesion molecules, E-cadherin, and β-catenin, which play essential roles in regulating embryonic development When the G6PD level is downregulated in cells, the cellular function of NOX is impaired, leading to the loss of redox signaling to induce the expressions of Smad3 and miR-200b. In such conditions, the expressions of E-cadherin and β-catenin are decreased, thus influencing the development of the embryo

The proposed mechanism concerning G6PD and the embryonic development of zebrafish (Fig. 1) is largely based on the novel findings of the current study, particularly that *G6PD* knockdown modulates the EMT process. Severe G6PD deficiency leading to embryonic lethality has been well documented. A mouse model has been used to demonstrate that G6PD is indispensable for the development of the embryo and placenta^9^. G6PD(−) hemizygous or homozygous embryos stop growing at E8.5 or E7.5, an important stage for establishing blood circulation in mice^9,23–25^. G6PD is required for placental development to support the trophoblast^9^, and it plays an embryoprotective role against the development of oxidative stress^11^. The zebrafish model demonstrates that severe *g6pd* knockdown results in the development of cardiac and kidney edema^18^. Notably, EMT plays a crucial role in embryogenesis^26^. E-cadherin, a key epithelial surface marker, is downregulated during EMT. The loss of E-cadherin leads to the breaking of cell junctions and contributes to the dissemination of neural crest cells during normal development^27,28^. E-cadherin expression is also involved in melanocyte development, especially in the migration from the neural crest^29,30^. β-Catenin is another essential protein in the EMT process, and it regulates cell movements, especially in gastrulation^31,32^. In the current study, *G6PD* knockdown impaired E-cadherin and β-catenin expression in cells and in the zebrafish model. Cardiac edema was observed in the *g6pd* morphants (as indicated by the arrowhead in Supplementary Figure 2b). *CDH1* and *G6PD* cRNA coinjection rescued the developmental defects of *g6pd* morphants in zebrafish. Taken together, these data provide strong support to the notion that *G6PD* knockdown impairs embryonic development by influencing the expression of adhesion molecules.

A key player in the proposed pathway involving G6PD and embryonic development is NOX signaling, which uses NADPH as a substrate to produce reactive oxygen species (ROS). NOX family members are responsible for redox signaling, and they have been discovered in the differentiation of various cell types such as neural crest cells^33^, cardiac muscle cells^34^, osteoblasts^35^, macrophages^36^, and renal profibrotic cells^37^. NOX-mediated redox signaling is also involved in cell-to-extracellular matrix interactions^38–40^ such as the anoikis molecular pathways. In the current study, NOX redox signaling modulated by G6PD was involved in the regulation of cell–cell interaction. Although NOX1 and NOX2 are the major isoforms in lung epithelial cells (as reported in another and in our previous study)^6,41^, NOX4 has also been implicated to be an essential mediator of Smad2/3 transcription factor activation in lung differentiation^42^. *G6PD* knockdown impaired p22_phox_ expression, the common subunit of NOX1, NOX2, NOX3, and NOX4, and thus decreased the expression of E-cadherin and β-catenin via redox-mediated signaling, possibly by modulating the Smad3/miR-200b pathway. Our data support the notion that G6PD status regulates embryonic development via redox signaling mediated through the NOX pathway. Additional studies are needed to clarify the specific roles of the various isoforms of NOX in redox signaling in different tissues during embryonic development.

The downstream targets in G6PD-mediated NOX signaling are Smad3 and miR-200b. *G6PD* knockdown decreased Smad3 expression concomitant with miR-200b downregulation (Fig. 2). The Smad pathway has a critical role in the expression of genes controlling embryonic stem cell differentiation^43–45^. Evidence indicates that Smad proteins inhibit EMT^43,46^. Smad3 modulates EMT in a TGF-β-independent manner through the regulation of miR-200 clusters^43^. MiR-200b, which is easily induced by oxidative stress, plays a critical role in modulating EMT, embryonic stem cell differentiation^47^, and the progression of cardiac function^48^. The expression of miR-200b induces the pluripotency of somatic cells through Oct4 and Sox2 upregulation^49^, the two major focuses of stem cell biology^50^. Taken together, G6PD plays a cytoregulatory role in producing a low concentration of ROS to affect the Smad3/miR-200b pathway through the modulation of NOX-mediated signaling during embryonic development. These data also support the concept that appropriate redox homeostasis is essential for controlling embryonic development.

Another novel observation in the current study highlights that *G6PD* knockdown downregulated β-catenin expression. β-Catenin has a dual function in gene transcription and cell−cell adhesion^51^. Alignment analysis was used to compare the protein sequences in humans (Homo sapiens) and zebrafish (Danio rerio), and >90% identical sequences over entire β-catenin lengths were discovered (Supplementary Table 2). The homological identities of G6PD and E-cadherin were 76.4 and 48.1% between these two species, respectively. β-Catenin is the key regulator for directing several developmental processes, especially via Wnt signaling, and the formation of different body regions, such as those involving cardiac physiology^52,53^. Furthermore, β-catenin has also been implicated in regulating the differentiation of mesodermal cell lineages such as macrophages and the asymmetric cell division of *C. elegans*. We noted that *G6PD* knockdown inhibited β-catenin expression, and cardiac edema occurred in the *g6pd* morphants of zebrafish. Moreover, *G6PD* knockdown can lead to the loss of asymmetric cell division in the embryos of *C. elegans*^12^ and can prohibit macrophage differentiation (our unpublished data). These data indicate that the G6PD status affects cell differentiation and embryonic development, partly via the β-catenin pathway.

Our novel findings indicate that G6PD plays a regulatory role in modulating EMT, which could have major clinical implications in carcinogenesis. Cancer cells exhibit increased glycolysis and pentose cycle activity compared with normal cells. These metabolic alternations were thought to arise from the damage to the respiratory mechanism, and cancer cells were thought to compensate for this defect by increasing glycolysis. An increase in G6PD activity is observed in most cancer cell lines, and this promotes cell proliferation. Accordingly, mutations leading to the overexpression of β-catenin are closely associated with cancer progression^54^. This may explain why G6PD upregulation is correlated with cancer progression through the upregulation of β-catenin signaling. By contrast, Anelisa et al. used proteomic analysis to demonstrate that tumorigenic glioblastomas have low G6PD protein expression and increased cell migration^55^. Our observations that *G6PD* knockdown impairs β-catenin expression but enhances cell migration are consistent with those of Anelisa et al.^55^. Because cancer progression is complicated with the participation of multiple factors, the role of G6PD in carcinogenesis needs to be further delineated.

In summary, our study demonstrated that G6PD is a common feature for regulating cell–cell interactions by modulating the expression of adhesion molecules. The current study also indicates that G6PD plays a cytoregulatory role in redox signaling by influencing the NOX pathway, which is critical to many cellular functions. The impaired NOX/Smad3/miR-200b axis is, in part, the mechanism through which *G6PD* knockdown impairs E-cadherin and β-catenin expression. Taken together, these data strongly suggest that in embryonic development, G6PD plays an essential role as an integral component of the NOX/Smad3/miR-200b axis.

## Materials and methods

### Materials

The following antibodies were used: anti-G6PD (Genesis Biotech Inc., New Taipei, Taiwan), anti-E-cadherin, anti-N-cadherin, anti-β-catenin (BD Biosciences, San Jose, CA, USA), anti-β-actin (GeneTex Inc., Irvine, CA, USA), alexa fluor 488 goat anti-mouse IgG, alexa fluor 594 phalloidin (Thermo Fisher Scientific, Waltham, MA, USA), anti-ZEB1, anti-Smad3 (Cell Signaling Technology, Danvers, MA, USA), anti-p22_phox_ (Abcam, Cambridge, UK), horseradish peroxidase (HRP)-conjugated anti-mouse IgG, and anti-rabbit IgG (Santa Cruz Biotechnology, Santa Cruz, CA, USA). Recombinant human TGF-β1 was purchased from PeproTech (Rocky Hill, NJ, USA). The *g6pd* targeting and nontarget Morpholinos were purchased from Gene Tools (Philomath, OR, USA).

### Cell culture

MDCK cells were kindly provided by Dr. Shih Sin-Ru at the Chang Gung University and were cultured in Dulbecco’s modified Eagle’s media containing 10% fetal bovine serum (FBS). Human adenocarcinoma epithelial cell lines (A549) were purchased from ATCC and cultured in DMEM supplemented with 10% FBS. These cells were maintained in a humidified atmosphere of 5% CO_2_ at 37 °C. Thawed cells from liquid nitrogen were used for within the first five passages.

### Plasmid construction and transduction by lentivirus system

pLKO.1.null-T and pLAS2w.Ppuro plasmids were purchased from the National RNAi Core Facility (Academia Sinica, Taipei, Taiwan). The mini-cassette encoding shRNA against G6PD, p22_phox_, or scramble gene include overhangs that are allowed cloning into pLKO.1.null-T. Scramble shRNA was used as a negative control. The sequence of G6PD shRNA cassettes were (sense) 5′- CCGGGGATACACACATATTCATCATCCTCGAGGATGATGAATATGTGTGTATCCTTTTT-3′ and (anti-sense) 5′-AATTAAAAAGGATACACACATATTCATCATCCTCGAGGATGATGAATATGTGTGTATCC-3′. The sequence of p22_phox_ shRNA cassettes were (sense) 5′- CCGGTACATGACCGCCGTGGTGAAGCTCGAGCTTCACCACGGCGGTCATGTATTTTT-3′ and (anti-sense) 5′-AATTAAAAATACATGACCGCCGTGGTGAAGCTCGAGCTTCACCACGGCGGTCATGTA-3′. The sequence of scramble shRNA cassettes were (sense) 5′-CCGGGCTCAACCTGTACAACATATTACTCGAGTAATATGTTGTACAGGTTGAGCTTTTT-3′ and (anti-sense) 5′-AATTAAAAAGCTCAACCTGTACAACATATTACTCGAGTAATATGTTGTACAGGTTGAGC-3′. The cDNA encoding human His-tagged G6PD (Accession No. NM_000402) or human SMAD3 (Accession No. NM_005902) was cloned into pLAAw.Ppuro plasmid (name as HTG6PD or SMAD3). Δ1339 was constructed by KOD plus polymerase (TOYOBO CO., Osaka, JP) using forward and reverse mutagenesis primers: (Forward) 5′-TGGACGTCTTCTGCAGGAGCCAGATGCACTTCG-3′ and (Reverse) 5′-CGAAGTGCATCTGGCTCCTGCAGAAGACGTCCA-3′. HEK293 cells were co-transfected with the packing plasmid (pCMV-ΔR8.91), pLKO.1 plasmids expressing shRNA/pLAS2w.Ppuro plasmids expressing cDNA, and envelope plasmid (pMD.G) to produce shRNA lentivirus according to instructions. The *G6PD* knockdown or overexpression were done by transducing with the recombinant lentivirus. The cDNA encoding human E-cadherin (Accession No.: NM_004360) was cloned into pCS2 + plasmid (Addgene, Cambridge, MA, USA).

### G6PD activity

G6PD activity was measured spectrophotometrically at 340 nm by the reduction of NADP^+^ in the presence of glucose-6-phosphate as previously described^5^. Briefly, cells or zebrafish embryos were collected and scraped in lysis buffer (50 mM Tris-HCl pH 7.4, 1% Triton X-100, 0.05% SDS, 150 mM NaCl, 1 mM EGTA and 1 mM NaF). Cell lysate was centrifugated and the supernatant was used in the assay. G6PD activity was analyzed by combining protein and assay buffer (50 mM Tris–HCl (pH 8), 50 mM MgCl_2_, 4 mM NADP^+^ and 4 mM glucose 6-phosphate).

### Western blot analysis

The cells and zebrafish embryos were scraped in lysis buffer, and centrifuged at 40,000 × g for 30 min at 4 °C to yield the extract. Samples were denatured, electrophoresed on 10% SDS-polyacrylamide gel, and transferred to PVDF membranes. The membranes were incubated overnight at 4 °C with an appropriate dilution of a primary antibody (1:1000) in Tris-buffered saline (50 mM Tris-HCl, 150 mM NaCl, 0.05% (w/v) Tween-20 (pH 7.4)) containing 5% (w/v) bovine serum albumin. Membranes were incubated with a 1:4000 dilution of anti-rabbit or anti-mouse-HRP antibody for 1.5 h. The immunoreactive bands were visualized by ECL reagents, with the signals captured by exposure to X-ray film. All experiments were performed independently at least in triplicate. (Unedited blots for all western blot experiments included in the supplementary data)

### Real-time PCR analysis

Total RNA of cells was isolated using Trizol reagent and reverse transcription was performed using the SuperScript III system (Invitrogen, Carlsbad, CA, USA). Primers were designed according to the sequences of human gene cDNAs and miRNA genes. The sequences of the primers used in reverse transcription and real-time PCR are listed in Supplementary Table 3. Real-time PCR was performed using the IQ™ SyBr Green Supermix kit on an IQ5 Real Time Thermal Cycler (Bio-Rad, Hercules, CA, USA). Relative fold expression values were determined using the ΔΔCt method. The gene expression levels were normalized to the level of the reference gene (*ACTB*) and miRNA expression level was normalized to the level of small nuclear RNA U6. All experiments were performed independently at least in triplicate.

### Immunofluorescence

A549 and MDCK cells were cultured in glass slides. After confluency, cells were fixed with 3.7% formaldehyde and permeabilized with 0.2% Triton X-100. Cells were incubated with mouse monoclonal anti-E-cadherin (1:50, BD Biosciences, San Jose, CA, USA), mouse monoclonal anti-β-catenin (1:200, BD Biosciences, San Jose, CA, USA), mouse monoclonal anti-N-cadherin (1:50, BD Biosciences, San Jose, CA, USA) overnight at 4 °C, and they were washed three times with PBS for 15 min each. Subsequently, the cells were incubated with alexa fluor 488 goat anti-mouse IgG secondary antibodies (1:100 for E- and N-cadherin and 1:400 for β-catenin, Thermo Fisher Scientific, Waltham, MA, USA) and with 2 µg/ml of Hoechst 33342 dye for nuclear staining for 60 min at room temperature. After incubation, the samples were rinsed 3 times with PBS for 15 min each. Finally, the samples were examined under an inverted fluorescence microscope (Olympus, Tokyo, JP).

### Migration assays and wound-healing experiment

In vitro migration assays, a total of 8 × 10^4^ cells in 200 μl serum-free Dulbecco's modified Eagle (DMEM) media were plated onto Millicell^®^ culture plate inserts (8 μm pore size; Merk Millipore, Darmstadt, Germany), and the lower chamber was immediately filled with 500 μl of DMEM media with 10% FBS as a chemoattractant. After 24 h of incubation in a humidified atmosphere containing 5% CO_2_ at 37 °C, the membranes were then fixed with methanol and stained by 0.2% crystal violet. Each individual experiment had triplicate inserts, and 5 microscopic fields were counted per insert. For wound-healing experiments, cells were plated in 6-well plates and cultured for confluency. Cells were serum-starved and scraped with a P200 tip (time 0), and pictures were taken at the 24 h time points then analyzed objectively using Image J. The percentage of wound closure at 24 h were measured by the size of the wound relative to that at 0 h in triplicate and the data were presented as mean ± SD.

### Ethics statement and fish maintenance

All experiments were performesd in accordance with standard guidelines for zebrafish work and approved by the Institutional Animal Care and Use Committee of Chang Gung University (IACUC approval number: CGU16-045). Tü (wild type) zebrafish embryos were purchased from the Zebrafish International Resource Center (Oregon) and were raised, maintained, and paired under standard conditions. The embryos were staged according to the number of somites, hours postfertilization, and days postfertilization^56^.

### RNA and morpholino injection

Capped RNA encoding the full coding sequences of human E-cadherin and G6PD were prepared as described previously^57^. Antisense morpholino (MO) oligonucleotides were purchased from Gene Tools (Philomath, OR, USA). The sequences of *g6pd* translation blocker and splice MOs were designed as the materials in previous study^18^. In brief, the *g6pd* MOs were combined in a 1:1 ration for delivery of 0.13 pmol for knockdown injection. Random MOs at 0.13 pmol were used for control injection. All injections were performed at the one- to two-cell stage and cRNAs or morpholinos were introduced into blastomeres.

### Statistical analysis

All data were expressed as means ± SD from replicated determinations. The student *t* test was used to compare the mean values from the two groups. Difference were regarded as significant the *p* value was <0.05.

## Supplementary information

Supplementary information

## References

[1] Cappellini MD, Fiorelli G (2008). Glucose-6-phosphate dehydrogenase deficiency. Lancet..

[2] Luzzatto L, Seneca E (2014). G6PD deficiency: a classic example of pharmacogenetics with on-going clinical implications. Br. J. Haematol..

[3] Wan GH, Tsai SC, Chiu DT (2002). Decreased blood activity of glucose-6-phosphate dehydrogenase associates with increased risk for diabetes mellitus. Endocrine..

[4] Gaskin RS, Estwick D, Peddi R (2001). G6PD deficiency: its role in the high prevalence of hypertension and diabetes mellitus. Ethn. Dis..

[5] Lin HR (2013). Proteome-wide dysregulation by glucose-6-phosphate dehydrogenase (G6PD) reveals a novel protective role for G6PD in aflatoxin B(1)-mediated cytotoxicity. J. Proteome. Res..

[6] Lin HR, Wu YH, Yen WC, Yang CM, Chiu DT (2016). Diminished COX-2/PGE2-Mediated Antiviral Response Due to Impaired NOX/MAPK Signaling in G6PD-Knockdown Lung Epithelial Cells. PLoS. ONE..

[7] Wu YH (2015). Glucose-6-Phosphate Dehydrogenase Enhances Antiviral Response through Downregulation of NADPH Sensor HSCARG and Upregulation of NF-kappaB Signaling. Viruses.

[8] Tang HY (2015). Inability to maintain GSH pool in G6PD-deficient red cells causes futile AMPK activation and irreversible metabolic disturbance. Antioxid. Redox. Signal..

[9] Longo L (2002). Maternally transmitted severe glucose 6-phosphate dehydrogenase deficiency is an embryonic lethal. EMBO. J..

[10] Yang HC (2013). *Glucose 6-phosphate deh*ydrogenase deficiency enhances germ cell apoptosis and causes defective embryogenesis in Caenorhabditis elegans. Cell death & disease.

[11] Nicol CJ, Zielenski J, Tsui LC, Wells PG (2000). An embryoprotective role for glucose-6-phosphate dehydrogenase in developmental oxidative stress and chemical teratogenesis. FASEB. J..

[12] Chen TL (2017). Impaired embryonic development in glucose-6-phosphate dehydrogenase-deficient Caenorhabditis elegans due to abnormal redox homeostasis induced activation of calcium-independent phospholipase and alteration of glycerophospholipid metabolism. Cell Death Dis.

[13] Nieto MA, Huang RY, Jackson RA, Thiery JP (2016). Emt: 2016. Cell.

[14] Feng XH, Derynck R (2005). Specificity and versatility in tgf-beta signaling through Smads. Annu. Rev. Cell. Dev. Biol..

[15] Yang J, Weinberg RA (2008). Epithelial-mesenchymal transition: at the crossroads of development and tumor metastasis. Dev. Cell..

[16] Mercado-Pimentel ME, Runyan RB (2007). Multiple transforming growth factor-beta isoforms and receptors function during epithelial-mesenchymal cell transformation in the embryonic heart. Cells. Tissues. Organs..

[17] Wu MY, Hill CS (2009). Tgf-beta superfamily signaling in embryonic development and homeostasis. Dev. Cell..

[18] Patrinostro X, Carter ML, Kramer AC, Lund TC (2013). A model of glucose-6-phosphate dehydrogenase deficiency in the zebrafish. Exp. Hematol..

[19] Babb SG, Marrs JA (2004). E-cadherin regulates cell movements and tissue formation in early zebrafish embryos. Dev. Dyn..

[20] Chilosi M (2017). Epithelial to mesenchymal transition-related proteins ZEB1, beta-catenin, and beta-tubulin-III in idiopathic pulmonary fibrosis. Mod. Pathol..

[21] Zhang J (2016). Inhibition of Glucose-6-Phosphate Dehydrogenase Could Enhance 1,4-Benzoquinone-Induced Oxidative Damage in K562 Cells. Oxid Med Cell Longev.

[22] Yang HC, Wu YH, Liu HY, Stern A, Chiu DT (2016). What has passed is prolog: new cellular and physiological roles of G6PD. Free. Radic. Res..

[23] Cox SJ, Gunberg DL (1972). Metabolite utilization by isolated embryonic rat hearts in vitro. J. Embryol. Exp. Morphol..

[24] Clough JR, Whittingham DG (1983). Metabolism of [14C]glucose by postimplantation mouse embryos in vitro. J. Embryol. Exp. Morphol..

[25] Ellington SK (1987). In vitro analysis of glucose metabolism and embryonic growth in postimplantation rat embryos. Development.

[26] Li X, Pei D, Zheng H (2014). Transitions between epithelial and mesenchymal states during cell fate conversions. Protein Cell.

[27] Carmona-Fontaine C (2008). Contact inhibition of locomotion in vivo controls neural crest directional migration. Nature..

[28] Stramer B (2010). Clasp-mediated microtubule bundling regulates persistent motility and contact repulsion in Drosophila macrophages in vivo. J. Cell. Biol..

[29] Nishimura EK, Yoshida H, Kunisada T, Nishikawa SI (1999). Regulation of E- and P-cadherin expression correlated with melanocyte migration and diversification. Dev. Biol..

[30] Mort RL, Jackson IJ, Patton EE (2015). The melanocyte lineage in development and disease. Development.

[31] Shimizu T (2005). E-cadherin is required for gastrulation cell movements in zebrafish. Mech. Dev..

[32] Larabell CA (1997). Establishment of the dorso-ventral axis in Xenopus embryos is presaged by early asymmetries in beta-catenin that are modulated by the Wnt signaling pathway. J. Cell. Biol..

[33] Lee JE, Cho KE, Lee KE, Kim J, Bae YS (2014). Nox4-mediated cell signaling regulates differentiation and survival of neural crest stem cells. Mol. Cells..

[34] Nadworny AS (2013). Nox2 and Nox4 influence neonatal c-kit(+) cardiac precursor cell status and differentiation. Am. J. Physiol. Heart. Circ. Physiol..

[35] Mandal CC (2011). Reactive oxygen species derived from Nox4 mediate BMP2 gene transcription and osteoblast differentiation. Biochem. J..

[36] Xu Q (2016). NADPH oxidases are essential for macrophage differentiation. J. Biol. Chem..

[37] Simone S (2012). BMP-2 induces a profibrotic phenotype in adult renal progenitor cells through Nox4 activation. Am. J. Physiol. Renal. Physiol..

[38] Chiarugi P, Giannoni E (2008). Anoikis: a necessary death program for anchorage-dependent cells. Biochem. Pharmacol..

[39] Groeger G, Quiney C, Cotter TG (2009). Hydrogen peroxide as a cell-survival signaling molecule. Antioxid. Redox. Signal..

[40] Paoli P, Giannoni E, Chiarugi P (2013). Anoikis molecular pathways and its role in cancer progression. Biochim. Biophys. Acta..

[41] Fink K, Duval A, Martel A, Soucy-Faulkner A, Grandvaux N (2008). Dual role of NOX2 in respiratory syncytial virus- and sendai virus-induced activation of NF-kappaB in airway epithelial cells. J. Immunol..

[42] Amara N (2010). NOX4/NADPH oxidase expression is increased in pulmonary fibroblasts from patients with idiopathic pulmonary fibrosis and mediates TGFbeta1-induced fibroblast differentiation into myofibroblasts. Thorax..

[43] Ahn SM (2012). Smad3 regulates E-cadherin via miRNA-200 pathway. Oncogene..

[44] Moustakas A, Souchelnytskyi S, Heldin CH (2001). Smad regulation in TGF-beta signal transduction. J. Cell. Sci..

[45] Massague J, Xi Q (2012). TGF-beta control of stem cell differentiation genes. FEBS Lett..

[46] Pohl M (2010). SMAD4 mediates mesenchymal-epithelial reversion in SW480 colon carcinoma cells. Anticancer. Res..

[47] Zhang J (2015). Retinoic Acid Induces Embryonic Stem Cell Differentiation by Altering Both Encoding RNA and microRNA Expression. PLoS. ONE..

[48] Feng B (2016). miR-200b Mediates Endothelial-to-Mesenchymal Transition in Diabetic Cardiomyopathy. Diabetes..

[49] Wang G (2013). Critical regulation of miR-200/ZEB2 pathway in Oct4/Sox2-induced mesenchymal-to-epithelial transition and induced pluripotent stem cell generation. Proc Natl Acad Sci U S A.

[50] Rizzino A (2013). Concise review: The Sox2-Oct4 connection: critical players in a much larger interdependent network integrated at multiple levels. Stem Cell.

[51] Brembeck FH, Rosario M, Birchmeier W (2006). Balancing cell adhesion and Wnt signaling, the key role of beta-catenin. Curr. Opin. Genet. Dev..

[52] Sokol SY (2015). Spatial and temporal aspects of Wnt signaling and planar cell polarity during vertebrate embryonic development. Semin. Cell. Dev. Biol..

[53] Grainger S (2016). Wnt9a Is Required for the Aortic Amplification of Nascent Hematopoietic Stem Cells. Cell Rep.

[54] Morin PJ (1999). beta-catenin signaling and cancer. Bioessays.

[55] Ramao A (2012). Changes in the expression of proteins associated with aerobic glycolysis and cell migration are involved in tumorigenic ability of two glioma cell lines. Proteome. Sci..

[56] Halpern ME (1995). Cell-autonomous shift from axial to paraxial mesodermal development in zebrafish floating head mutants. Development.

[57] Chung PC (2011). Zebrafish Her8a is activated by Su(H)-dependent Notch signaling and is essential for the inhibition of neurogenesis. PLoS. ONE..

