# Supplementary material for: Glucose-6-phosphate dehydrogenase is indispensable in embryonic development by modulation of epithelial-mesenchymal transition via the NOX/Smad3/miR-200b axis
Source: Cell Death Dis. 2018 Jan 9;9(1):10. doi: 10.1038/s41419-017-0005-8 (PMC5849038; doi:10.1038/s41419-017-0005-8)

**Supplementary information**

**Title :** Glucose-6-phosphate dehydrogenase is indispensable in embryonic development by modulation of epithelial-mesenchymal transition via the NOX/Smad3/miR-200b axis

Materials and methods

**Materials.** G6PD (L-008181-02-0020) and control (D-001810-10-20) siRNA were purchased from GE Healthcare Dharmacon (Lafayette, CO, USA). ZEB1 and control siRNA were purchased from Sigma Aldrich (Merck KGaA, Darmstadt, Germany). Pharmacological inhibitor of miR200b, miRCURY LNA inhibitor targeting hsa-miR-200b (Exiqon; 4100278-001) and control (Exiqon; 199006-001) were purchased from EXIQON (QIAGEN, Hilden, Germany).

**Transfection of siRNAs.** The A549 cells (5 × 10^5^) were seeded on six-well plates and transfected 24 h later with plasmids using LF2000 (Invitrogen). During transient transfection with siRNA, the cells were transfected with 10 nM siRNA (ON-TARGETplus Human G6PD siRNA and ON-TARGETplus Non-targeting Pool). The nontargeting siRNA was used as a control for nonspecific effects of transfected siRNA. After transfection for 72 h, the cells were harvested for real-time PCR.

**Transfection of LNA.** The cells (5 × 10^5^) were seeded on six-well plates and transfected 24 h later with LNA using LF2000 (Invitrogen). During transient transfection with LNA, the cells were transfected with 50 nM LNA. After transfection for 72 h, the cells were harvested for Western blot analysis.


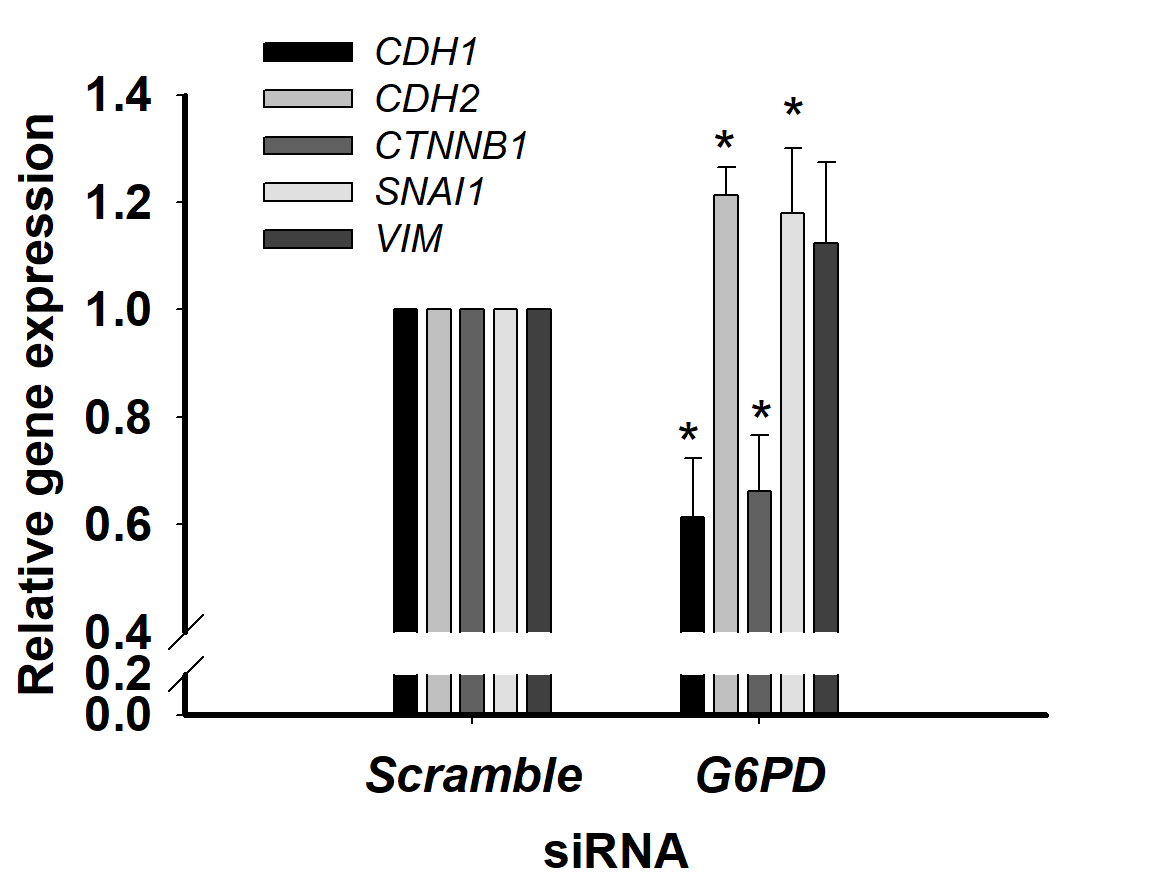


**Figure S1** The expressions of EMT markers were altered by the G6PD status. Compared with control cells transfected with scramble siRNA, a significant decrease in the gene expressions of *CDH1* and *CTNNB1* and an increase in *CDH2* and *SNAI1* expression in *G6PD* knockdown A549 cells were observed through Real-time PCR analysis. The transcript level of target gene was determined through real-time PCR, normalized to *ACTB*, and calculated relative to the scramble group (set to 1). Results are presented as mean ± SD from three independent experiments (*p < 0.05). The gene expression of *TWIST1* was undetectable in this condition.


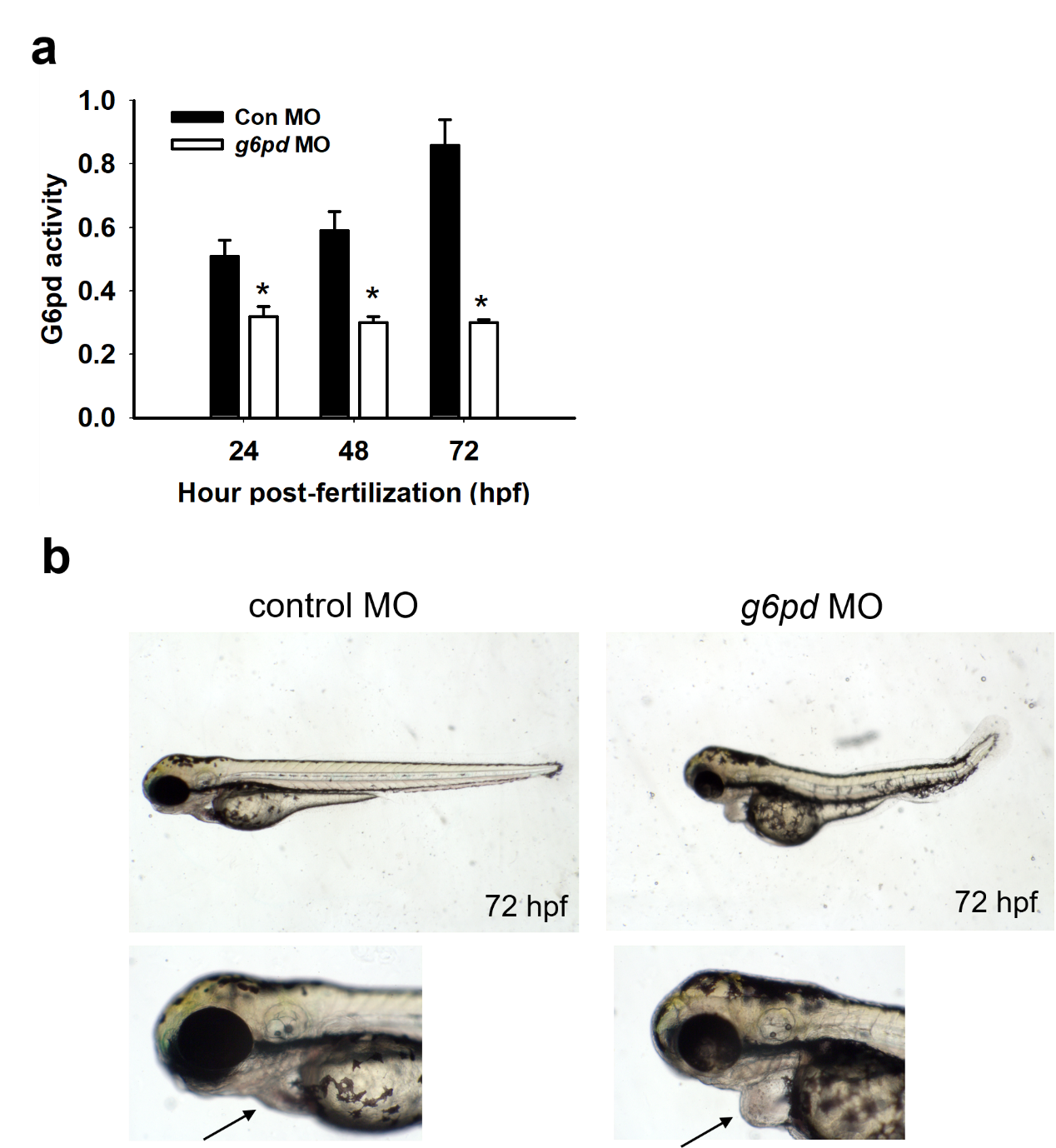


**Figure S2** The kinetics of G6pd activity at 24, 48, and 72 h postfertilization (hpf) are shown. **(a)**The kinetic data of control MO revealed that G6pd activity was increased during zebrafish embryonic development. However, g6pd MO injection reduced G6pd activity. **(b)** Cardiac edema was observed in the g6pd morphants (as indicated by the arrowhead).

**
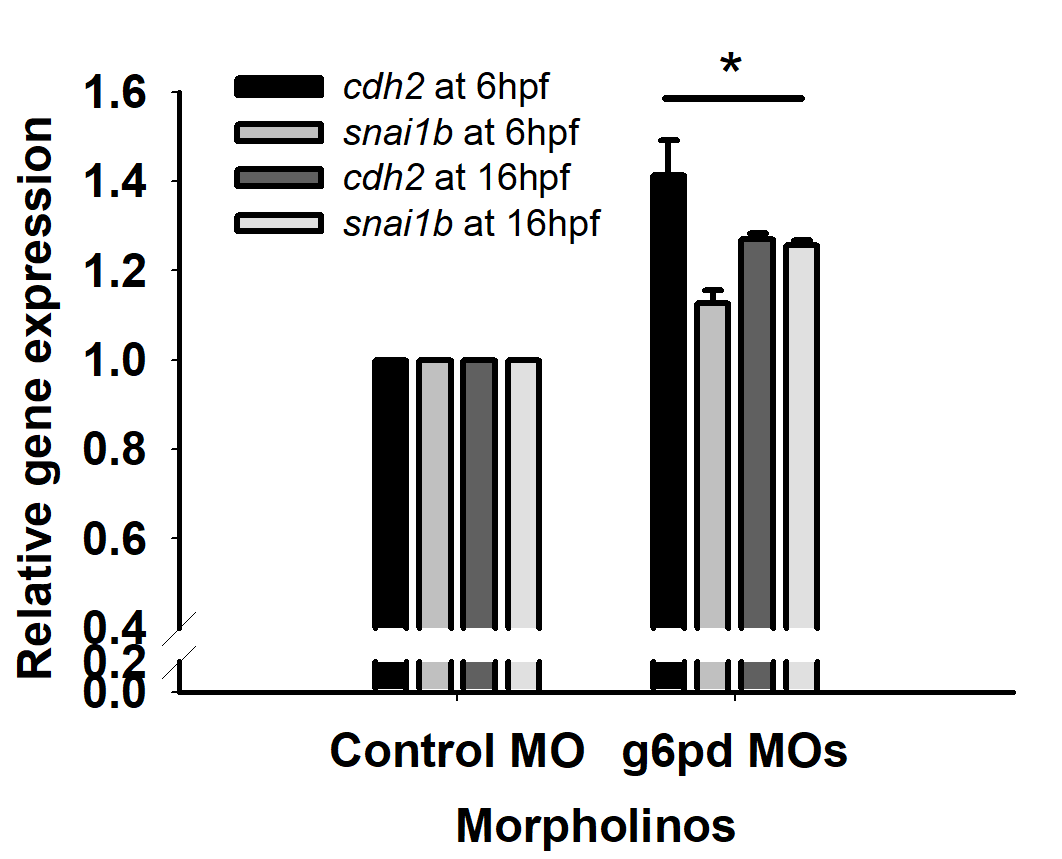
**

**Figure S3** The expressions of EMT markers were altered by the G6PD status in zebrafish embryos (6 and 16 hpf). The *cdh2* and *snai1b* gene expressions in zebrafish embryos injected with control or *g6pd* MOs were determined through real-time PCR, normalized to *actb2*, and calculated relative to the control group (set to 1). Results are presented as mean ± SD from three independent experiments (*p < 0.05).


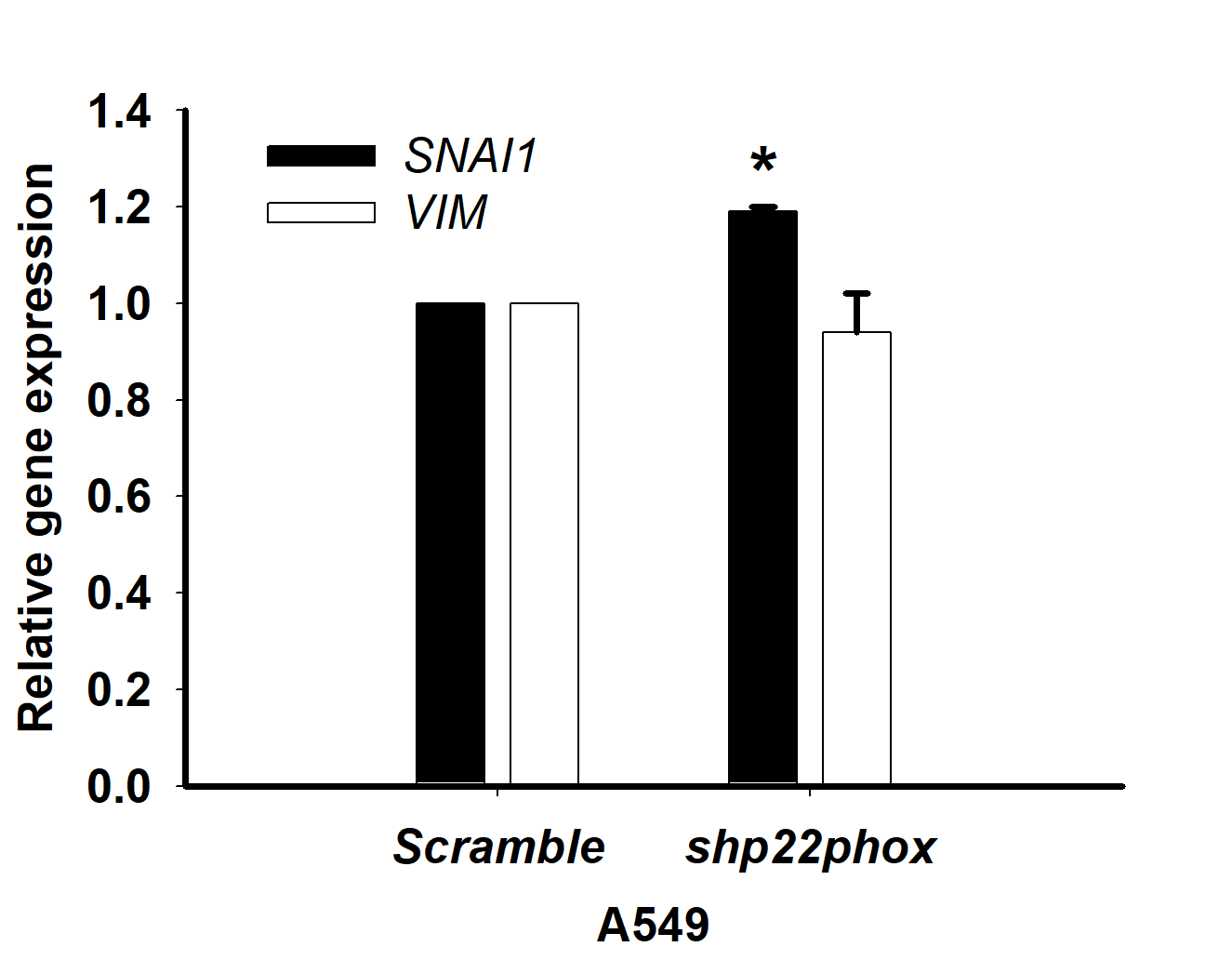


**Figure S4** The expressions of *SNAI1* and *VIM* were analyzed in *shp22_phox_* knockdown and scramble control A549 cells. The transcript level of target gene was determined through real-time PCR, normalized to ACTB, and calculated relative to the scramble group (set to 1). Results are presented as mean ± SD from three independent experiments (*p < 0.05). The gene expression of TWIST1 was undetectable in this condition.


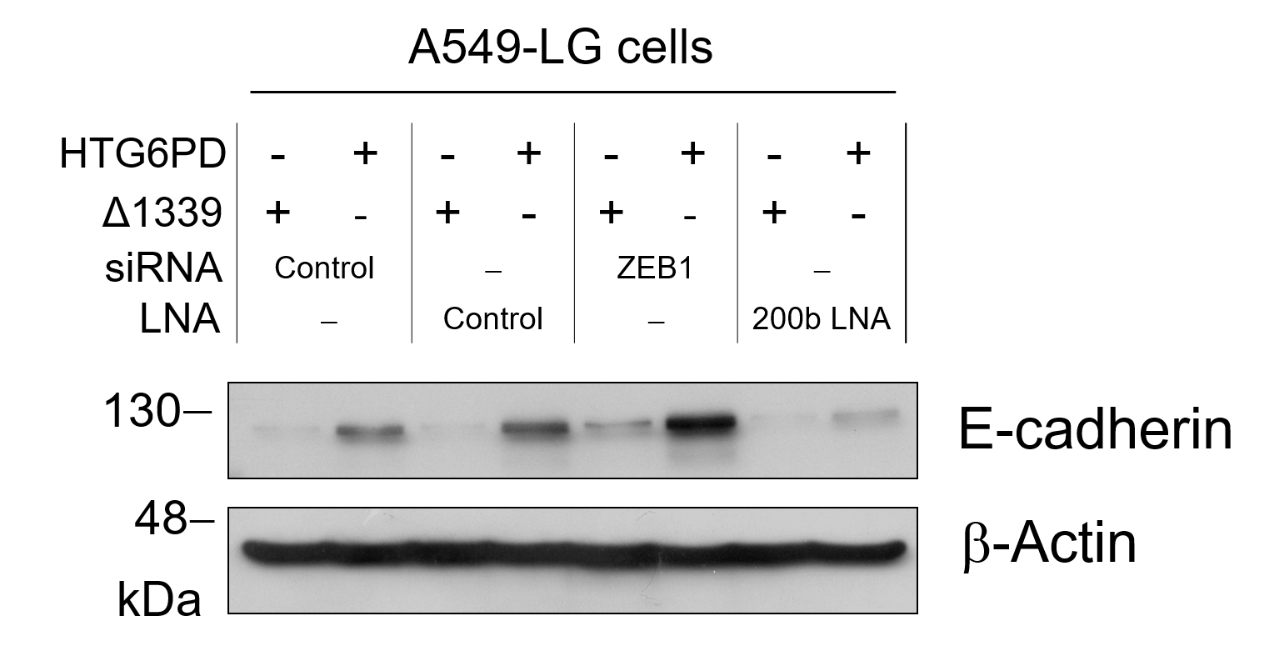


**Figure S5** The expression of E-cadherin was altered by the ZEB1 siRNA or miR-200b LNA in *Δ1339* or *HTG6PD* A549-LG cells. The protein expression of E-cadherin was observed through Western blot analysis. β-Actin was used as the loading control.


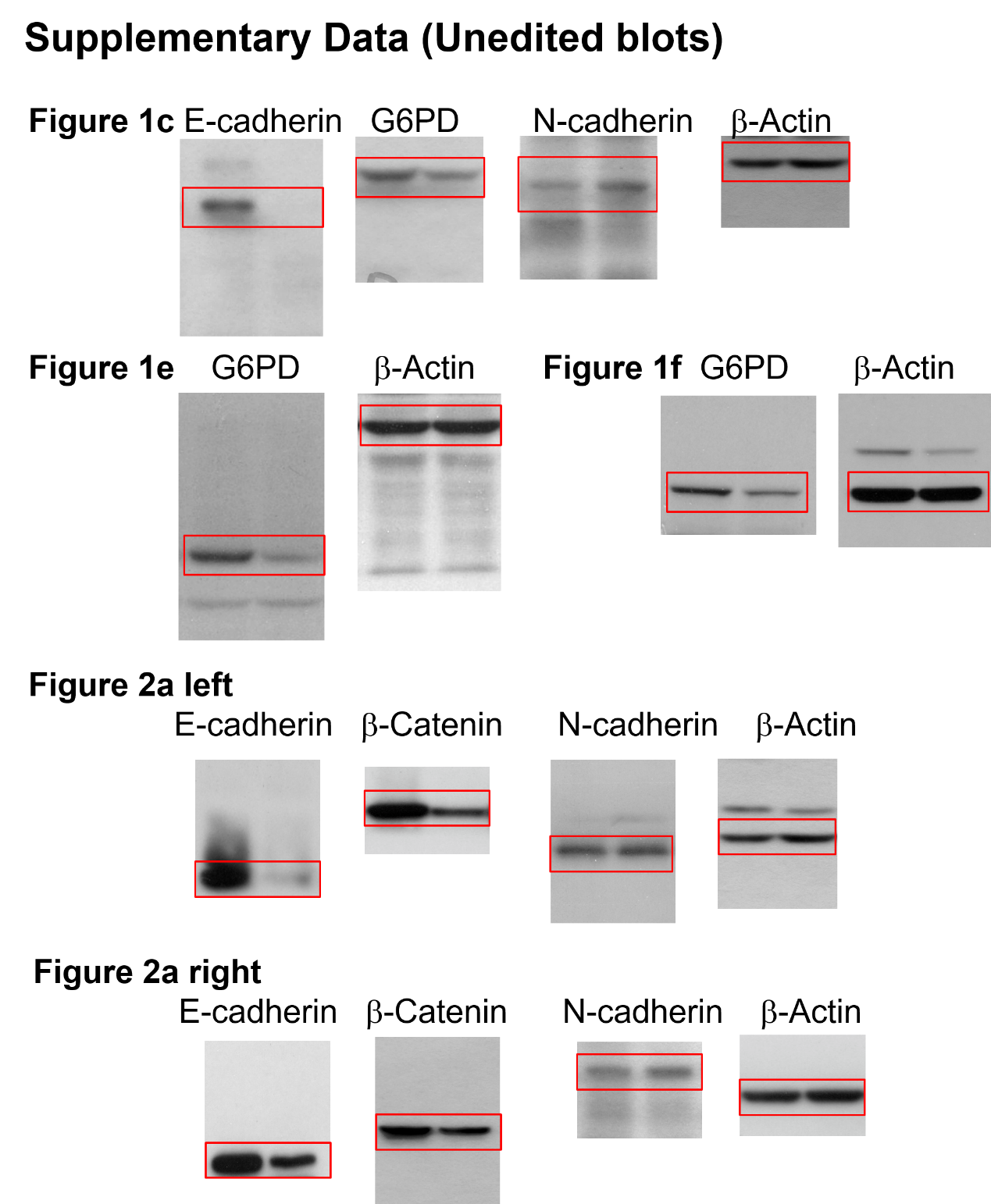

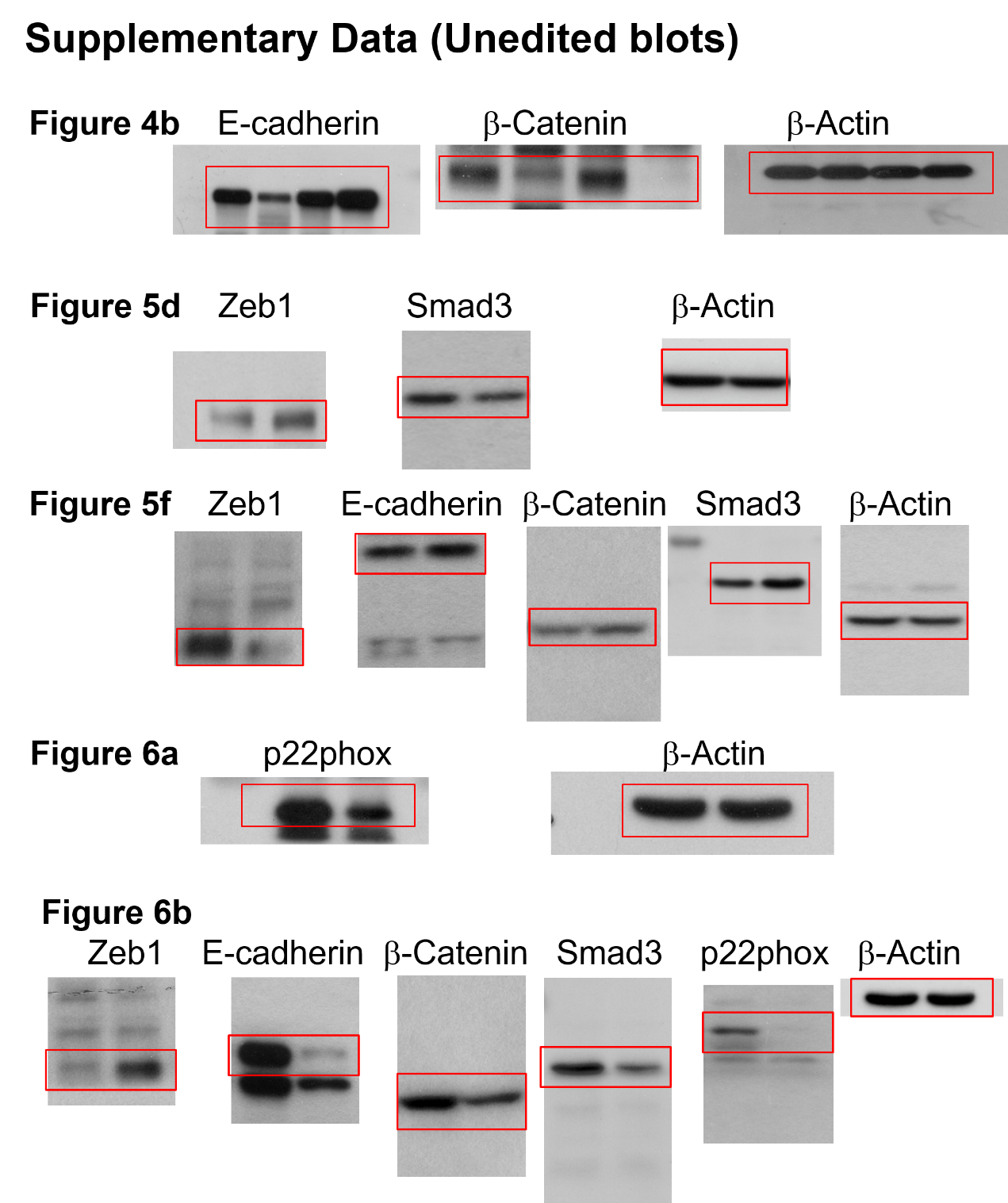

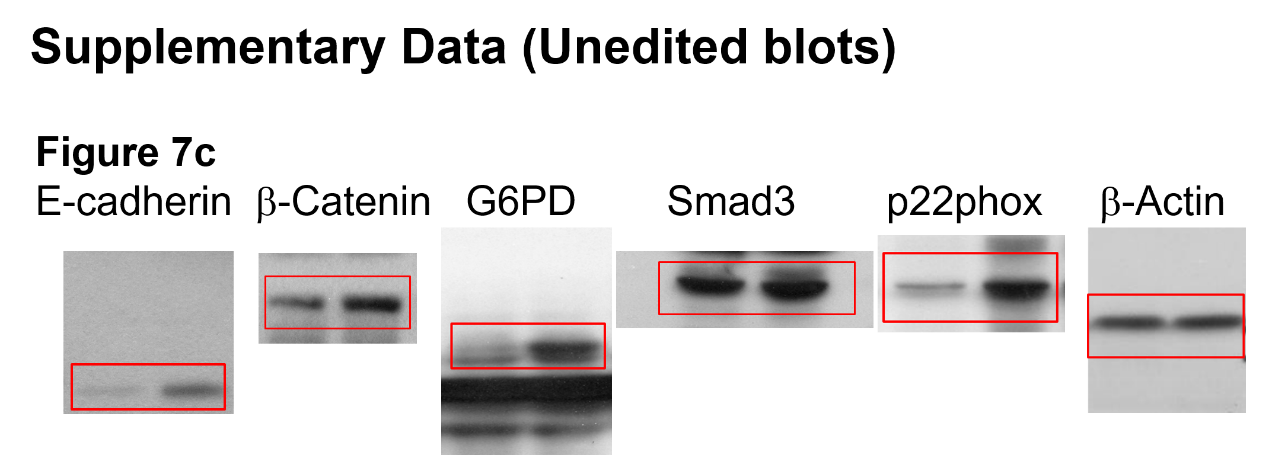


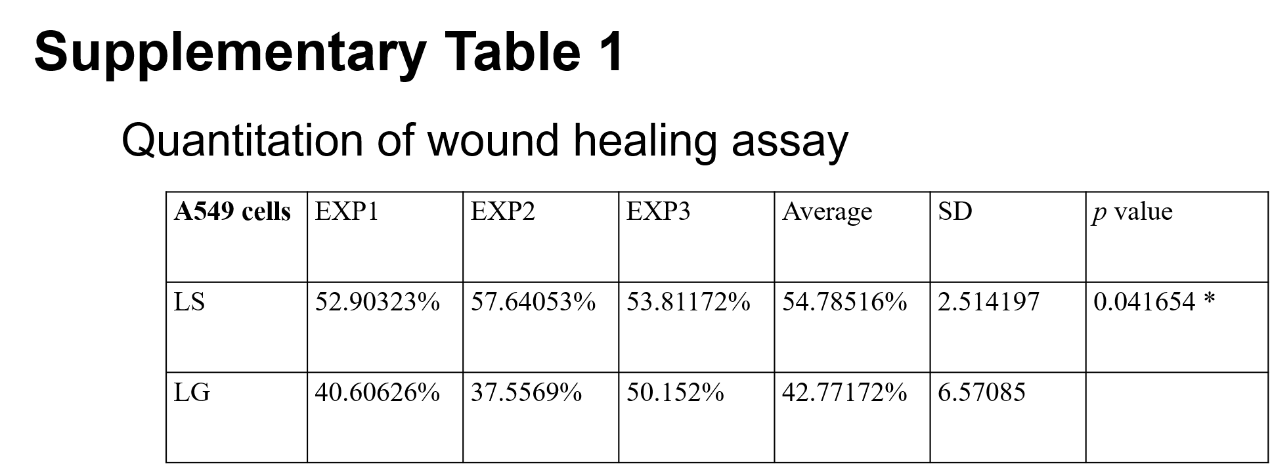


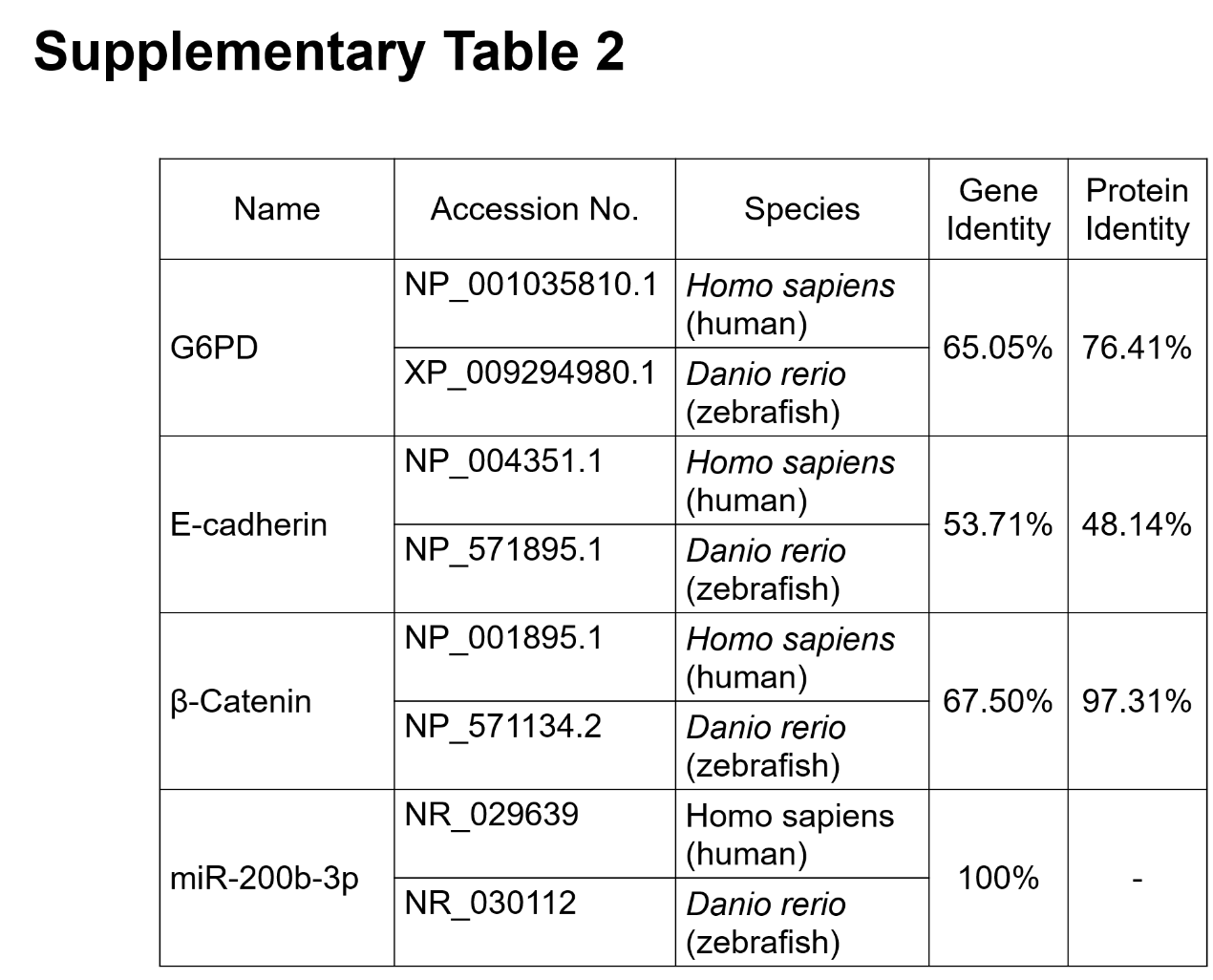


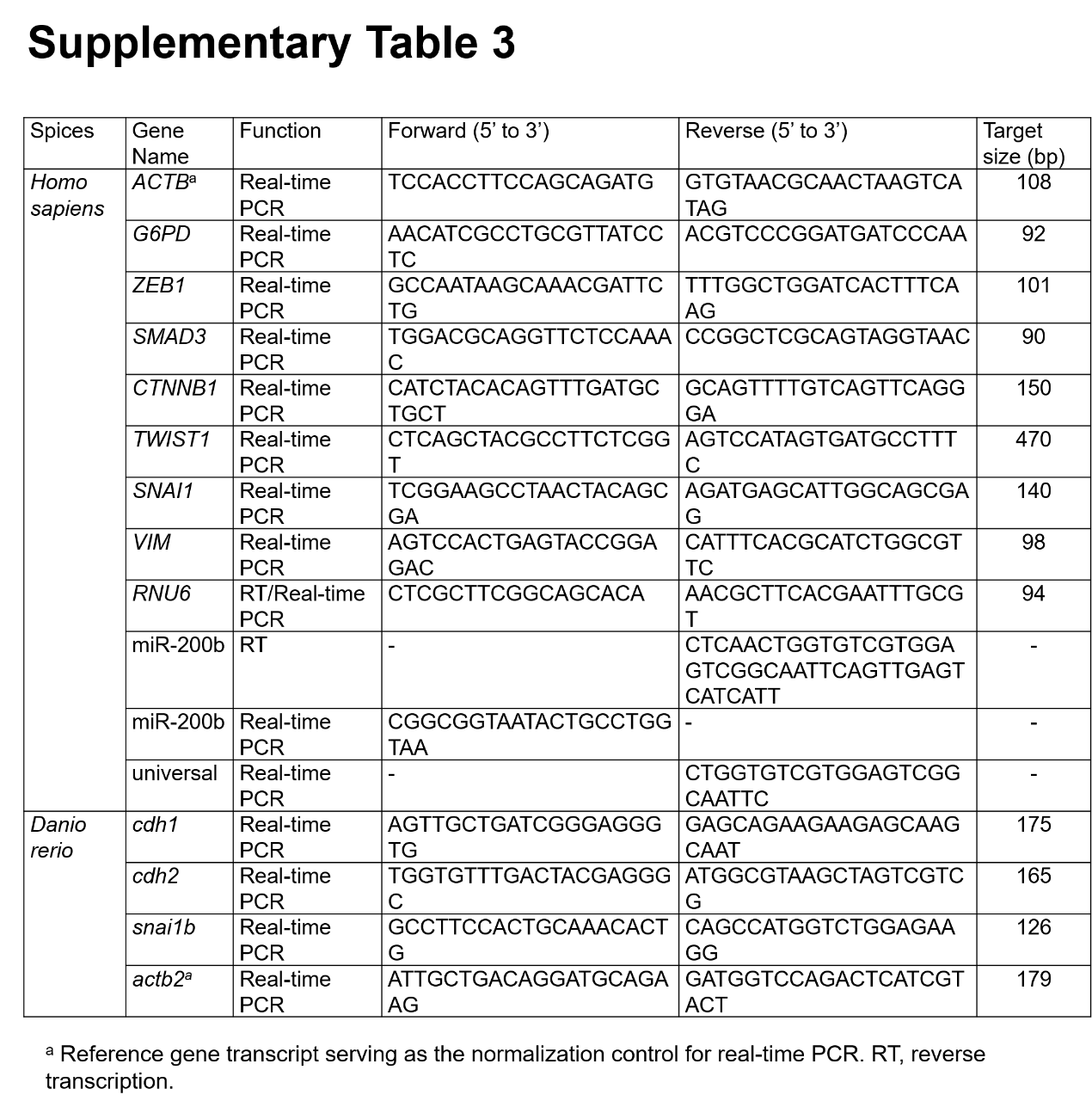

Supplement: Supplementary file 1 — Supplementary information [file 41419_2017_5_MOESM1_ESM.docx]
